# Docking and Molecular Dynamics Simulation‐Based Analysis of Advanced Small‐Molecule Kinase Inhibitors Identified pre‐*let*‐*7* miRNA Binders

**DOI:** 10.1002/cbic.202500421

**Published:** 2025-08-26

**Authors:** Soma Roy, Yang Liu, Peng Wu

**Affiliations:** ^1^ Chemical Genomics Centre Max Planck Institute of Molecular Physiology Otto‐Hahn Str. 11 44227 Dortmund Germany; ^2^ Department of Chemical Biology Max Planck Institute of Molecular Physiology Otto‐Hahn Str. 11 44227 Dortmund Germany; ^3^ Faculty of Chemistry and Chemical Biology TU Dortmund University Otto‐Hahn Str. 6 44227 Dortmund Germany

**Keywords:** docking, *let‐7* miRNA, molecular dynamics simulation, RNA‐binding small molecule, small‐molecule kinase inhibitor (SMKI)

## Abstract

RNAs play crucial roles in various cellular actions, and the uncontrolled expression or improper folding of RNAs is a cause of many diseases. Certain oncogenic phenotypes stem from the overexpression of regulatory microRNAs that contain secondary structural elements. Thus, targeting disease‐related microRNAs with small molecules is a potential therapeutic strategy that has attracted growing interest. To probe the RNA‐binding chemical space held in advanced small‐molecule therapeutics, in this work, we screened the 78 FDA‐approved small‐molecule kinase inhibitors (SMKIs) as representatives of the most advanced kinase inhibitors for their binding affinity with pre‐*let‐7* miRNA via a combination of computational methods and biophysical measurement. The best‐ranked SMKIs based on docking scores were subjected to molecular dynamics simulation studies, followed by analysis of different computational parameters. Collectively, it provided reliable information on the binding affinity for the top‐performed SMKI in the formation of SMKI–miRNA complexes with pre‐*let‐7.* Furthermore, the identification of the predicted most promising SMKI–miRNA interactions was validated by microscale thermophoresis, measuring direct binding affinities. This study evaluating the binding landscapes of the 78 FDA‐approved SMKIs with pre‐*let‐7* miRNA served as an example highlighting the necessity to characterize the biological targets of SMKIs, many of which are FDA‐approved cancer agents, at the transcriptomic level with RNAs. The study also illustrates the possibility that the interaction with RNA targets may contribute to the observed biological and clinical performance of these approved SMKIs.

## Graphical abstract

An integrated computational approach evaluating the binding between pre‐*let*‐*7* microRNA and small‐molecule kinase inhibitors (SMKIs) revealed varied pre‐*let*‐*7* binding affinities among the advanced SMKIs. The results justified the characterization of potential RNA targets for kinase inhibitors and small molecules with protein targets in general.

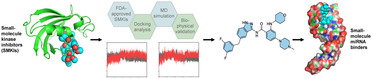

## 1 Introduction

Targeting RNA with small molecules has emerged as an appealing approach that distinguishes it from the traditional protein‐centered approach for the development of therapeutics for human diseases.^[^ ^1^ ^]^ In parallel with the evolving understanding of the important functions that RNAs play in physiological and diseased cellular states, an increasing number of small molecules have been identified as binders or biological modulators for structured RNAs.^[^ ^2^ ^]^ About 90% of the human genome is transcribed into RNAs, the majority of which are associated with non‐protein‐coding functions.^[^ ^3^ ^]^ For example, the small endogenous non‐coding miRNAs (18–25 nucleotides long) play regulatory roles in gene expression at the post‐transcription level by binding messenger RNA (mRNA) transcripts. The aberrant expression of miRNAs is a distinctive signature for a range of human cancers.^[^ ^4^ ^]^ It has been 30 years since the discovery of the first miRNA (characterized as a small non‐coding RNA in *C. elegans*) as a regulator of the lin‐14 protein expression.^[^ ^5^ ^]^ The essential developmental gene *lethal*−7 (*let*−7) in *C. elegans* was eventually discovered as one of the first miRNAs. Although the ambiguous function of miRNAs is a largely unexplored and exciting research topic, the tumor‐suppressing function of mature *let‐7* plays in downregulating *MYC*, *RAS*, and other oncogenes is well established.^[^ ^6^ ^]^ Therefore, small‐molecule RNA binders could potentially be used to modulate *let‐7* biogenesis and maturation.

**Figure 1:**
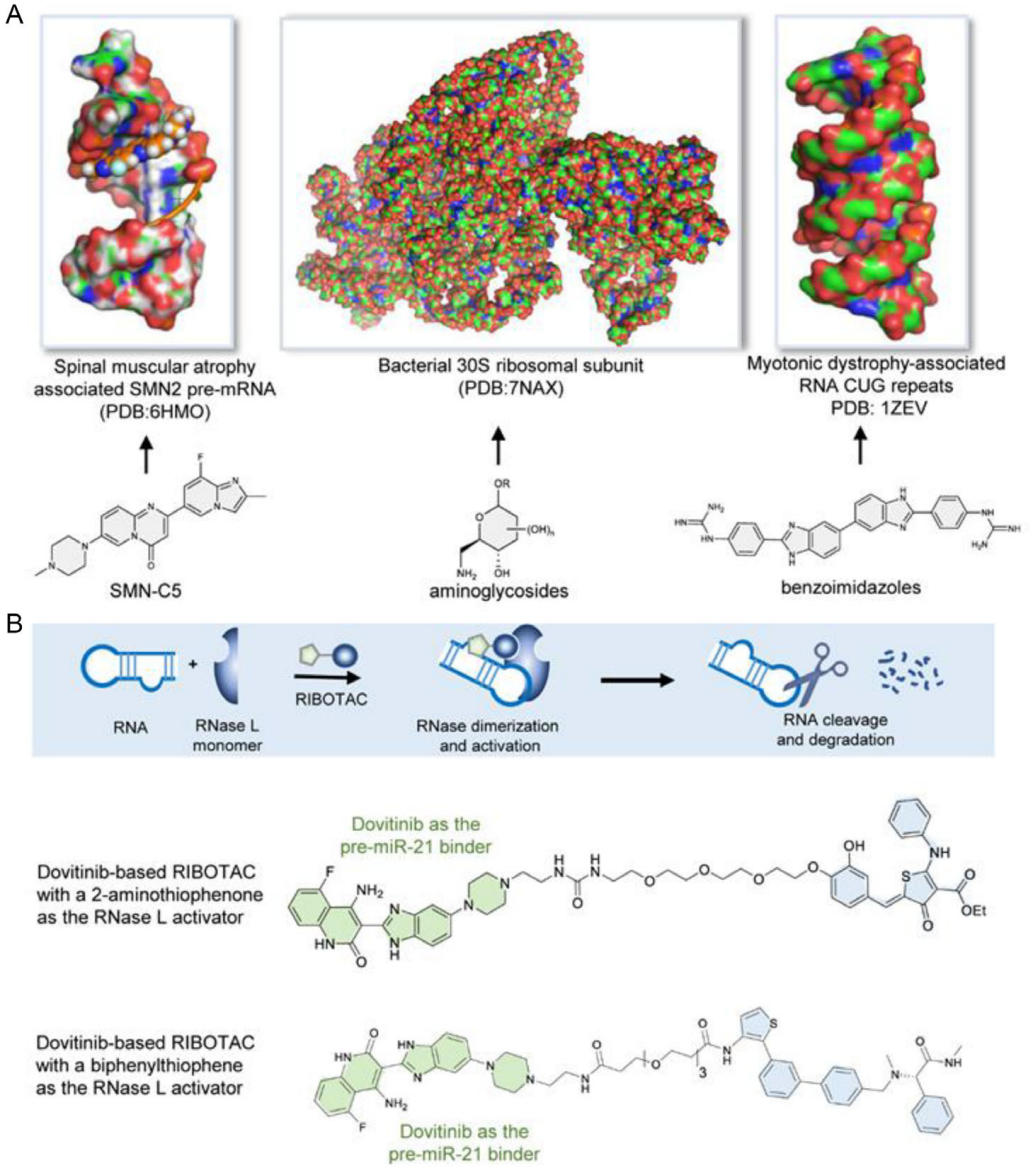
Chemotypes of RNA‐binding small molecules. A) Reported examples of small‐molecule binders to different RNA species, including SMN‐C5 as an alternative splicing modulator targeting *SMN2* pre‐mRNA associated with spinal muscular atrophy, aminoglycosides as rRNA binders, and benzoimidazoles as binders to RNA CUG repeats associated with myotonic dystrophy. B) Ribonuclease‐targeting chimeras (RIBOTACs) that recruit the latent ribonuclease (RNase L) to the proximity of an RNA substrate to achieve targeted degradation. Kinase inhibitors, such as dovitinib, have been used as the pre‐miR‐21 binder to assemble dovitinib‐based RIBOTACs for miR‐21 degradation by coupling to a small‐molecule 2‐aminothiophenone or biphenylthiophene RNase L activator.

Although the chemotypes of small molecules and chemical space required to target RNAs are yet to be thoroughly defined, insightful structures have been reported from encouraging scattered examples,^[^ ^7^ ^]^ such as the pyrimidinones including SMN‐C5 and risdiplam as *SMN2* pre‐mRNA alternative splicing modulators,^[^ ^8^ ^]^ aminoglycosides as rRNA binders,^[^ ^9^ ^]^ and benzoimidazoles as binders to RNA repeats (**Figure** **1A**).^[^ ^10^ ^]^ Meanwhile, an emerging trend lies in the identification of small‐molecule kinase inhibitors (SMKIs) with ‘off‐target’ RNA‐binding activities, such as the receptor tyrosine kinase inhibitors dovitinib and regorafenib, both of which showed binding to the precursor miRNA‐21.^[^ ^11^ ^]^ Of particular note, the RNA‐binding affinity of dovitinib has found practical utility in chemical biology applications in the assembly of proximity‐inducing ribonuclease‐targeting chimeras (RIBOTACs), which have been demonstrated for the targeted degradation of a range of different RNA species via the recruitment and activation of the latent ribonuclease (RNase L) by a small‐molecule activator (e.g. 2‐aminothiophenone or biphenylthiophene) (Figure 1B).^[^ ^12^ ^]^

Whereas once deemed as ‘undruggable’ given the highly conserved ATP‐binding pockets and high physiological ATP concentrations, kinases are now among the most successful drug targets developed to date,^[^ ^13^ ^]^ with no less than 78 SMKIs having been approved by the US FDA for both cancer and non‐cancer indications.^[^ ^14^ ^]^ The recent revelation of the above‐mentioned RNA‐binding and ‐targeting activities of advanced SMKIs, including the FDA‐approved SMKIs, is a highly intriguing but barely systematically studied direction in the kinase‐targeting field, which could contribute directly to providing diverse chemotypes that are eagerly sought after to address biological and therapeutic applications associated with various RNA‐targeting strategies.

In this context, associated with our current interest in targeting the LIN28–*let‐7* interactions with small molecules,^[^ ^15^ ^]^ we wondered if the current collection of the 78 FDA‐approved drugs as the most advanced SMKIs bears binding potency to the structured element of precursor *let‐7* (pre‐*let‐7*). Herein, we screened the 78 FDA‐approved SMKIs for binding with the pre‐*let‐7* miRNA leveraging computer‐aided drug design approaches combined with biophysical validation.^[^ ^16^ ^]^ Such methods including docking and molecular dynamics (MD) simulation have promoted the study of binding modes and interactions between small‐molecule ligands and biomacromolecule targets^[^ ^17^ ^]^ and facilitated the identification of corresponding binding ligands combined with experimental validations.^[^ ^18^ ^]^ We evaluated the binding of the 78 SMKIs to pre‐*Let‐7* miRNA by first performing HDOCK analysis. The best‐evaluated SMKIs were then subjected to MD simulation to acquire information on potential modes of binding, energetics, solvent‐accessible surface area (SASA), and hydrogen‐bond interactions. The binding affinities of the SMKIs towards pre‐*let‐7* were validated by the following biophysical measurement using microscale thermophoresis. The results provided molecular‐level information on the SMKI–pre*‐let‐7* interactions and indicated an understudied area of evaluating SMKI–RNA interactions and RNA‐binding potential of SMKIs that could be associated with the biological performance of SMKIs.

## 2 Results and Discussion

### 2.1 Docking and MD Simulation Identified SMKIs as pre‐*let‐7* Binders

**Figure 2:**
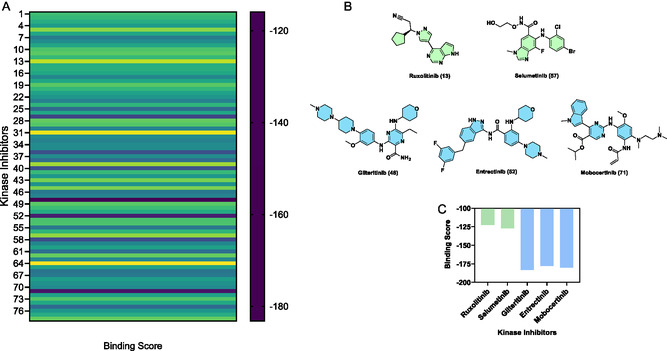
Docking analysis based on the 78 FDA‐approved SMKIs. A) Distribution of the docking scores predicted for the SMKIs’ binding with the pre‐*let‐7* miRNA. Each number on the *Y*‐axis indicates an SMKI numbered in Table S1, Supporting Information. The structures for the full 78 SMKIs are provided in Figure S1, Supporting Information. B) The two least potent and three most potent SMKIs as pre‐*let‐7* binders based on the docking analysis. C) Docking scores for the five SMKIs are shown in panel B.

We started with the docking analysis between the 78 FDA‐approved SMKIs and the pre‐*let‐7* miRNA using the pre‐*let‐7* structure extracted from the reported LIN28–*let‐7* complex (PDB ID: 5UDZ).^[^ ^19^ ^]^ As our goal was to evaluate the potential binding interaction between SMKIs and pre‐*let*‐*7* miRNA, we excluded the LIN28 protein in the docking analysis. HDOCK server was used to dock the SMKIs with the pre‐*let‐7*.^[^ ^20^ ^]^ The docking scores for the 78 SMKIs are shown in **Figure** **2A** (Figure S1 and Table S1, Supporting Information), which revealed that the 78 SMKIs exhibited largely varied scores with a cohort of SMKIs showing promising scores that warranted follow‐up evaluations. Among the 78 SMKIs, the lowest docking scores ranging from −115.92 to −126.95 were predicted for tirbanibulin, cobimetinib, ruxolitinib and selumetinib, while the highest docking scores ranging from −177.75 to −183.16 were predicted for entrectinib, mobocertinib, and gilteritinib (Table S1, Supporting Information). Due to the readily availability of ruxolitinib and selumetinib, we used these two compounds as the negative comparisons in the following experimental tests. The structure of ruxolitinib and selumetinib with the low docking scores and the predicted top‐three most potent binders (gilteritinib, entrectinib, and mobocertinib) with the highest docking scores are depicted in Figure 2B with the calculated docking score shown in Figure 2C. Based on the docking analysis, we proceeded to perform the MD simulation study using the top‐three ranked compounds (gilteritinib, entrectinib, and mobocertinib) to further probe the insights into the potential binding modes.

**Figure 3:**
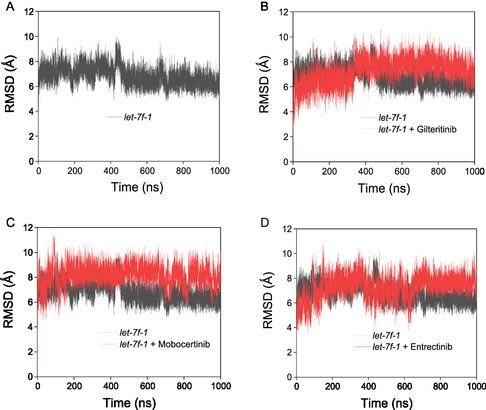
MD simulation plots. A) pre‐*let‐7* alone without SMKIs. B–D) SMKI–pre‐*let‐7* complexes with gilteritinib, mobocertinib, and entrectinib, respectively, in the duration of 1000ns.

**Figure 4:**
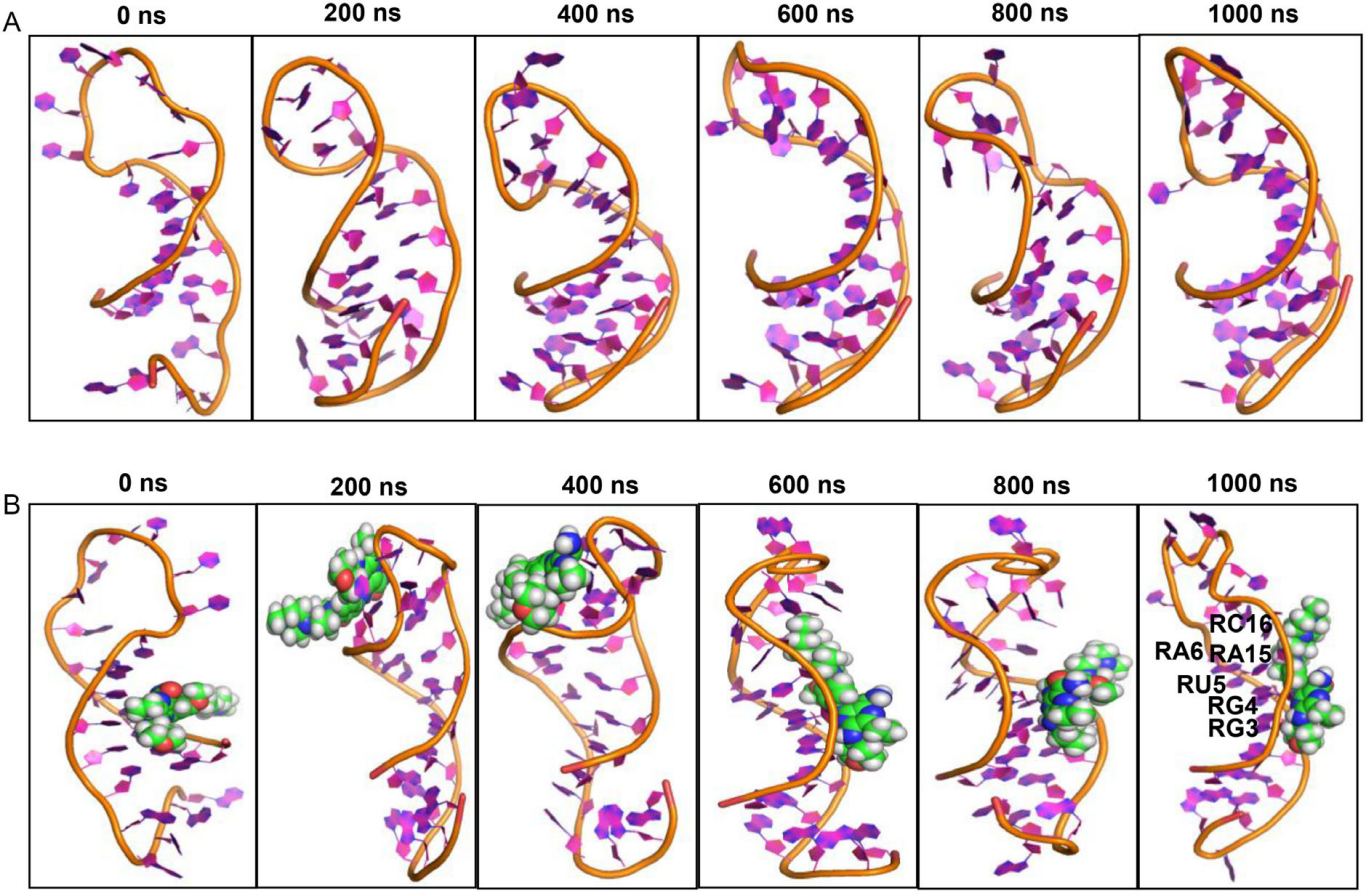
MD snapshots at different time points. A) pre‐*let*−7 miRNA alone without SMKIs. B) Gilteritinib–pre‐*let*−7 complex.

**Figure 5:**
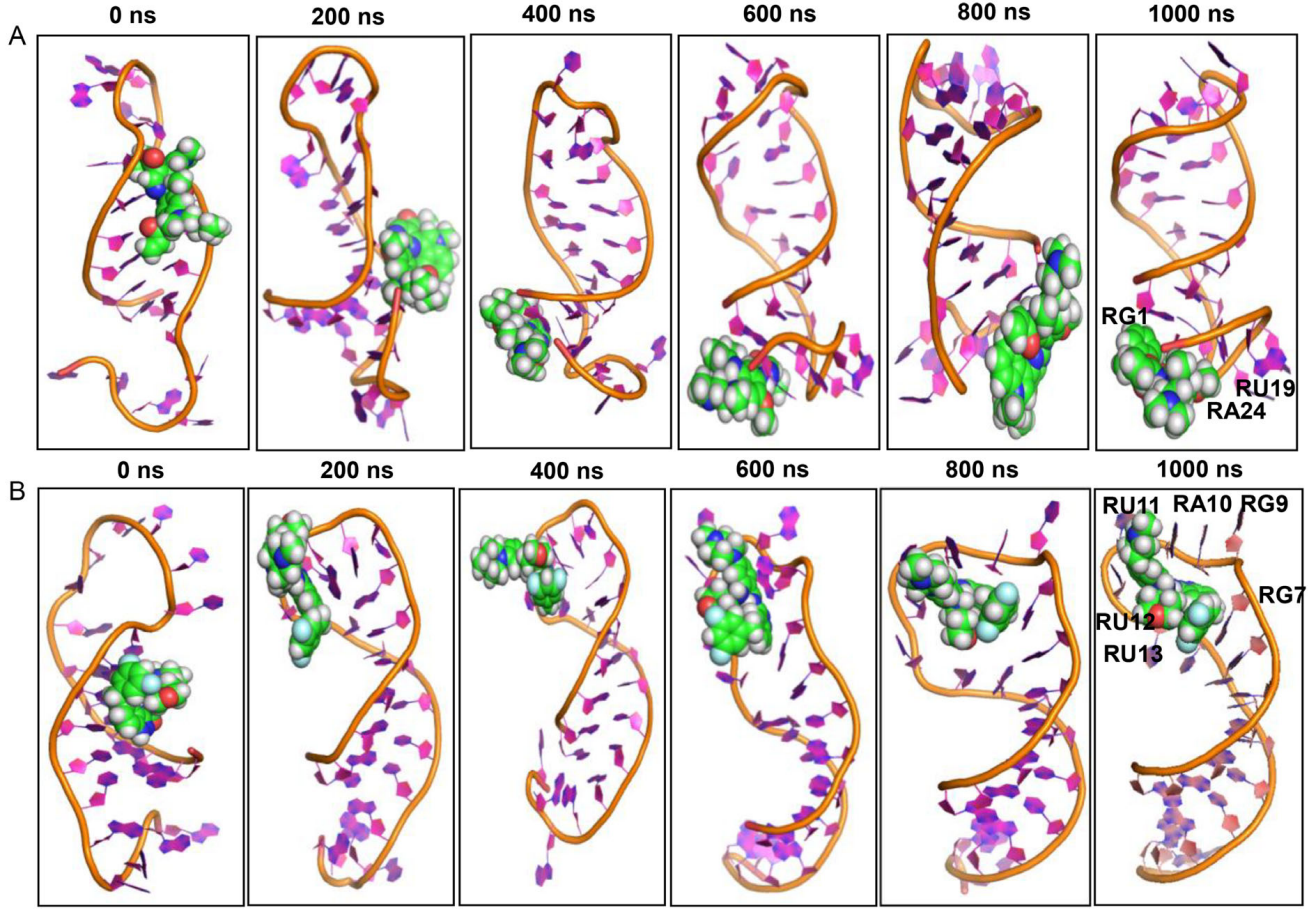
MD snapshots at different time points. A) Mobocertinib–pre*‐let‐7* complex. B) Entrectinib–pre*‐let‐7* complex.

**Figure 6:**
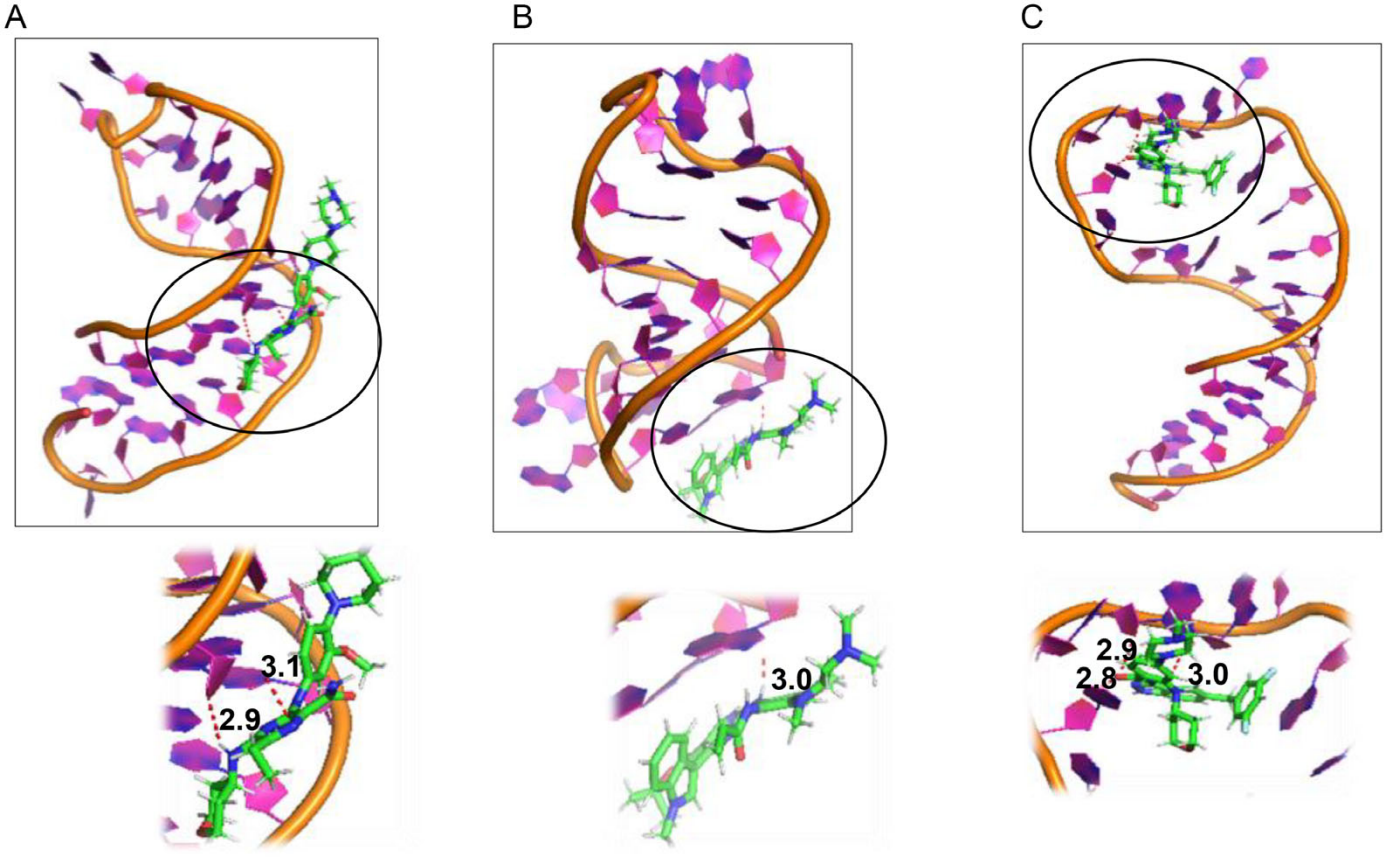
H‐bonding interactions of SMKI–miRNA complexes in the final 100 ns of the 1000ns simulation time for A) gilteritinib, B) mobocertinib, and C) entrectinib. The circled portions are enlarged below indicated the measure H‐bonding distances (Å).

The SMKI–miRNA complexes were subjected to MD simulation to monitor the interaction over a period of 1000ns. As a comparison, the miRNA alone was subjected to the MD simulation for the specified time (**Figure** **3**). Based on the RMSD plots, it is noteworthy that both the miRNA alone and SMKI‐miRNA complexes were stable over the simulation period. We analyzed the RMSDs (20 fs per each time step) of the ligands throughout the simulation (Figure S2, Supporting Information). The RMSD profiles suggested that although the values are moderately elevated, both the free ligands and the ligand‐RNA complexes preserved their structural coherence throughout the duration of the simulation. The MD snapshots of the pre‐*let‐7f‐1* miRNA alone and the SMKI–miRNA complexes at different time points are shown in **Figure** **4**‐ **5**, which overall indicated that the dynamic structure of miRNA resulted in the observed fluctuation of the SMKI–RNA complexes. The structure of the pre‐*let‐7* miRNA remained stable throughout the simulation time (Figure 4A). The binding of gilteritinib stabilized pre‐*let‐7* in the groove surrounded by RG3, RG4, RU5, RA6, RA15, and RC16 (Figure 4B). In the simulation period of 1000ns, mobocertinib bound with the bases of RG1, RU19, and RA24 (Figure 5A), and entrectinib interacted with the loop of the pre‐*let‐7* miRNA surrounded by the bases of RG7, RG9, RA10, RU11, RU12, and RU13 (Figure 5B). Additionally, these observations arise from the fluctuations of the bases, as illustrated in the RMSF vs. nucleotide plot (Figure S3, Supporting Information). To analyze the stabilizing factors in the SMKI–miRNA interaction, we plotted the hydrogen bond occurrences. As shown in **Figure** **6** (and Table S2, Supporting Information) for the SMKI–miRNA complexes involving gilteritinib, mobocertinib, and entrectinib, respectively, the potential formation of the hydrogen bonds between pre*‐let‐7* bases and the respective SMKIs contributed to the stability of the SMKI‐miRNA complexes.

### 2.2 Solvent Accessible Surface Area Evaluating the Compactness of the SMKI–miRNA Complex

**Figure 7:**
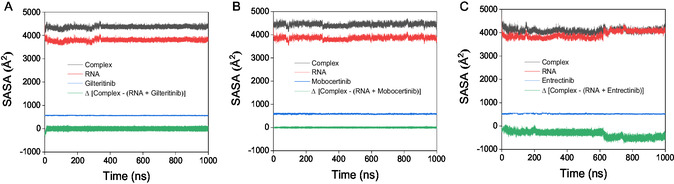
SASA plots of the SMKI–miRNA complex in comparison with that of pre‐*let‐7* miRNA and gilteritinib (A), mobocertinib (B), and entrectinib (C).

In the following evaluation, the SASA was calculated for the pre‐*let‐7* miRNA, SMKI–miRNA complexes, and the bound SMKI extracted from the complexes. As shown in **Figure** **7**, the ΔSASA [complex‐(RNA + ligand)] values were slightly negative for the mobocertinib–miRNA complex. The RNA‐ligand complexes consisting of gilteritinib and entrectinib ligands exhibited higher negative ΔSASA values as compared to that of mobocertinib. In the case of the entrectinib–miRNA complex, ΔSASA dropped at 620 ns and eventually stabilized after the simulation period of 745 ns. Overall, the observed slight decrease in the SASA values for the SMKI–miRNA complexes in the simulation duration manifested the probable compactness of the miRNA and SMKI upon the complex formation.

### 2.3 Radius of Gyration (Rg) Indicating Stability of the Complexes

Based on the calculated radius of gyration values (Figure S4, Supporting Information), it can be observed that the RNA‐SMKI complexes maintain stability while RNAs interact with the SMKIs. This also suggests that the RNA remains compactly folded and structurally stable throughout the simulation period, highlighting the degree of compactness.

### 2.4 *π*‐*π* Interaction Energies are Observed to be Comparable for the Three Compounds

The *π*‐*π* interactions play crucial roles in determining binding interactions between small molecules and structured RNA elements by influencing their stability, dynamics, molecular recognition, etc. We determined the intrinsic *π*‐*π* interactions (base stacking) for gilteritinib, mobocertinib, and entrectinib from the MD trajectories and the values are in the range of −1.3 to −2.1 kcal mol^−1^ (Table S3, Supporting Information). Thus, the *π*‐*π* interaction energies are observed to be comparable for the three compounds.

### 2.5 Free Energy Calculations Indicated Thermodynamically Favored Formation of the SMKI–miRNA Complex

The feasibility of the complex formation between SMKIs and miRNA is another practical concern. We calculated the energies of the native miRNA and the SMKI–miRNA complexes using MM/GBSA free energy analysis with a set of 100 snapshots acquired from the last 10 ns of the final production run of the MD simulations. It was noticed that Δ*G* values were −22.2, −20.4, and −28.13 kcal mol^−1^ for the gilteritinib–RNA, mobocertinib–RNA, and entrectinib–RNA, respectively (**Table** **1**). These negative magnitude values suggested the formation of stable ligand‐RNA associations. Furthermore, the entropic contribution of the ligands for binding to miRNA in the complexes was calculated. The total entropic costs (T*Δ*S) of the three complexes were observed to be −24.8, −19.7, and −22.8 kcal mol^–1^ for gilteritinib‐RNA, mobocertinib‐RNA, and entrectinib‐RNA, respectively (**Table** **2**). These data demonstrated that the association of the selected SMKIs with miRNA for the complex formations was favored thermodynamically.

**Table 1:** Contribution to the binding free energies (kcal⋅mol^–1^) acquired from the MM/GBSA calculation for the gilteritinib–miRNA, mobocertinib–RNA, and entrectinib–RNA complexes.

Entry	*E*_VDW_	*E*_ELEC_	*E*_GB_	*E*_SURF_	Δ*G*_GAS_	Δ*G*_SOLV_	Δ*G*_TOTAL_
Gilteritinib–miRNA	−42.1 ± 0.3	−19.1 ± 0.7	43.5 ± 0.8	−4.5 ± 0.02	−61.2 ± 0.8	39.0 ± 0.8	−22.2 ± 0.4
Mobocertinib–miRNA	−35.8 ± 0.3	13.3 ± 0.9	4.7 ± 0.8	−2.6 ± 0.01	−22.4 ± 0.8	2.1 ± 0.8	−20.4 ± 0.3
Entrectinib–miRNA	−52.7 ± 0.4	5.2 ± 0.4	23.7 ± 0.5	−4.3 ± 0.03	−47.6 ± 0.6	19.4 ± 0.5	−28.13 ± 0.4

**Table 2:** Different components of the entropic contributions (TΔS; kcal⋅mol^−1^) of the SMKI–miRNA complexes involving gilteritinib, mobocrtinib, and entrectinib.

Entry	Translational	Rotational	Vibrational	Entropic contribution
Gilteritinib–miRNA	−13.3 ± 0.0	−11.6 ± 0.0	0.07 ± 0.0	−24.8 ± 1.1
Mobocertinib–miRNA	−13.3 ± 0.0	−11.5 ± 0.0	5.1 ± 0.3	−19.7 ± 0.3
Entrectinib–miRNA	−13.3 ± 0.0	−11.4 ± 0.0	1.9 ± 0.7	−22.8 ± 0.6

### 2.6 Ligand Strain Energy Validates the Docking and Simulation Processes

The strain potential energies of the ligands were calculated to assess ligand deformation free energies and evaluate docking quality using the best‐docked structures. After extracting the lowest‐energy conformers from both bound and water‐solvated simulations (Figure S2 and S5, Supporting Information), global strain energies were determined following a reported procedure. As shown in Table S4, Supporting Information, gilteritinib and entrectinib exhibit lower strain potential energies (2.6 and 2.8 kcal mol^–1^, respectively) compared to mobocertinib (4.3 kcal mol^–1^), likely due to the higher number of rotatable bonds in the latter. Nevertheless, these values fall within acceptable cutoff ranges, further validating the docking and simulation processes.

Lower‐ranked ligands such as ruxolitinib and selumetinib were each subjected to 1000ns MD simulations, during which they displayed higher RMSD values than the top‐ranked compounds. Ruxolitinib and selumetinib exhibited lower binding energies compared to the top‐ranked compounds (gilteritinib, entrectinib, and selumetinib), further supporting the higher binding efficiency and stability of the top candidates (Figure S6, Supporting Information).

### 2.7 Experimental Validation of the Binding Affinity via Biophysical Measurement

**Figure 8:**
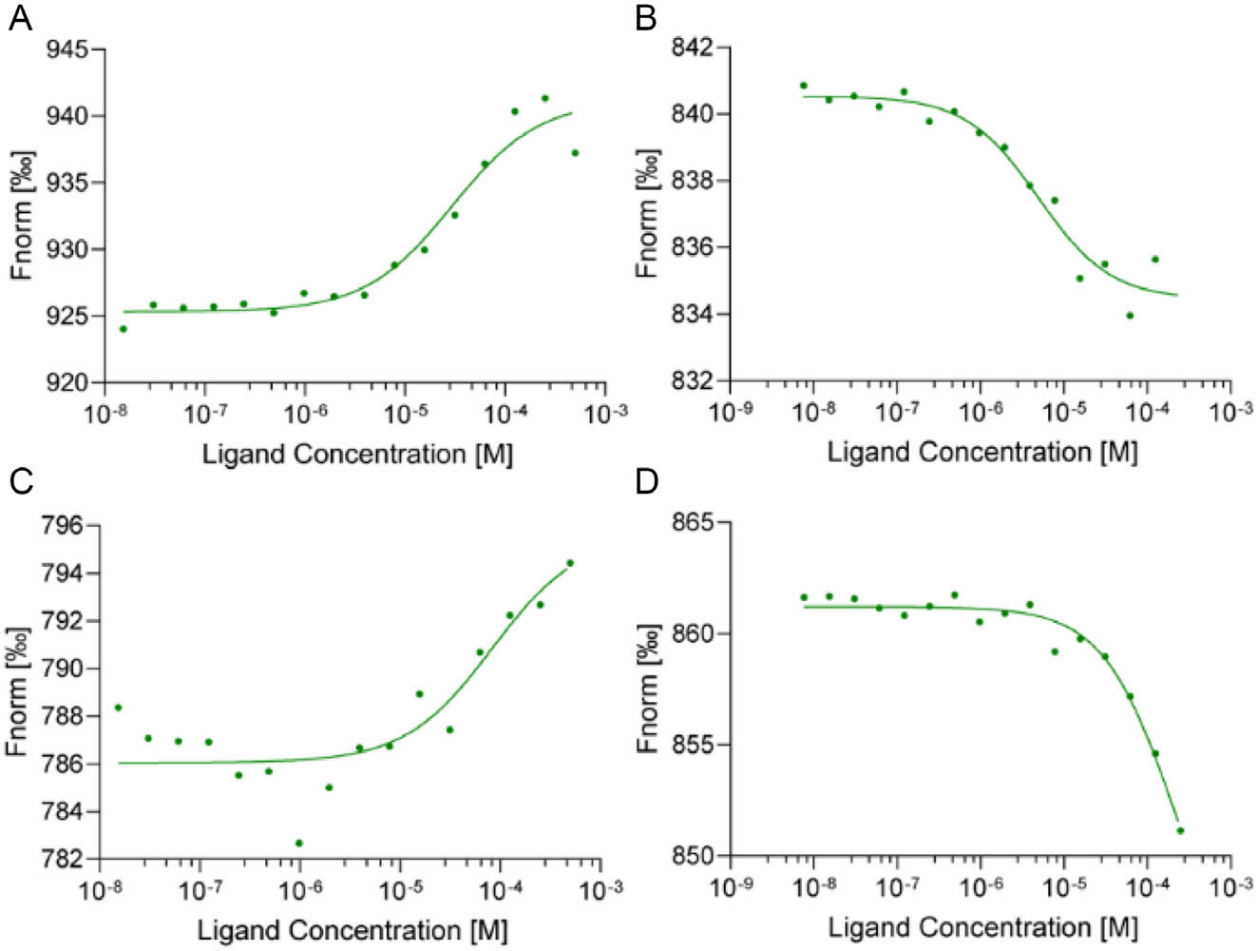
Experimental evaluation of selected SMKIs’ binding affinity towards pre*‐let‐7* miRNA via thermophoresis analysis by MST. A) gilteritinib (*K*_d_: 29.4 µM). B) Entrectinib (*K*_d_: 5.0 µM). C) Ruxolitinib (*K*_d_: 82.4 µM). D) Selumetinib (*K*_d_: 217 µM).

The results obtained from the above MD simulation‐based analysis were further validated by the experimental measurement of the binding affinities using microscale thermophoresis (MST). We used pre‐*let*‐7f‐1 for computational docking due to the availability of resolved structural data, while pre‐*let*‐7a‐1, a well‐established expression model, was used in the MST experiment. Among the top‐ranked SMKIs, gilteritinib and entrectinib exhibited *K*_d_ values of 29.4 and 5.0 µM, respectively (**Figure** **8**). The least potent compounds ruxolitinib and selumetinib showed *K*_d_ values of 82.4 and 217 µM respectively. Further, the effective *let‐7* binders gilteritinib and entrectinib did not exhibit any dose‐responsive graphs after the addition of miRNA21 in the MST measurement, which showed the preferential binding of the SMKIs towards pre‐*let7* miRNA as compared to miRNA21 (Figure S7, Supporting Information). To provide support for the observed specificity, we performed HDOCK‐based docking of gilteritinib and entrectinib with miRNA21 (PDB ID: 5UZT), which yielded lower binding scores (−142.2 and −127.9, respectively) compared to their docking with pre‐*let*−7, aligning with the biophysical data and confirming the predicted selectivity. In a word, the biophysical experimental data validated the predicted results described above in the computational sections.

### 2.8 Conclusion and Future Perspective

Whereas kinase‐targeting and SMKIs have achieved unparalleled success in treating human diseases, especially cancers, the interactions between SMKI and RNAs is an intriguing but rarely studied subject that could have a direct impact on many aspects of both kinase‐targeting and RNA‐targeting research. To reveal the potential RNA‐binding chemotypes folded in the chemical space covered by kinase inhibitors, we studied the binding affinity of the 78 FDA‐approved SMKIs toward miRNA, exemplified by pre‐*let‐7*, in this study. Our findings indicate that top‐ranked SMKIs, based on computational docking and MD analyses, specifically gilteritinib and entrectinib, are binders to pre‐*let*‐*7*, with their binding affinities validated in a biophysical method using MST. In comparison, lower‐ranked compounds such as ruxolitinib and sulometinib exhibit weaker binding affinities. On the one hand, it is reported that small‐molecule RNA binders can be both biologically active modulators for RNA biogenesis and biologically inactive inert binders.^[^ ^12^ ^b]^ The identified ‘off‐target’ effects of RNA‐binding activities of these SMKIs, as exemplified by gilteritinib and entrectinib, hold readily applicable utility in chemical biology, such as the assembly of the SMKIs as potential RNA‐recruiting components into RIBOTACs to achieve targeted cleavage of specific RNAs. That being said, on the other hand, it is important to note that the RNA‐binding affinities for the evaluated SMKIs are weak (moderate micromolar potency) in comparison with their reported kinase‐binding affinities and kinase inhibitory activities (nanomolar potency); thus, the ultimate question whether such RNA‐binding properties for the approved and marketed kinase inhibitors contribute to their pharmacological and clinical performance, either in positive or negative manners, warrants further extensive investigations. Overall, the study provides a straightforward evaluation pipeline that can be practically applied to screening SMKIs towards other structured RNAs as the first filter to identify RNA‐binding SMKIs that can either be developed as new RNA‐targeting chemotypes or be applied in the assembly of proximity‐inducing bifunctional molecules targeting RNAs, including RIBOTACs.

## 3 Experimental Section

3.1

3.1.1

##### Docking Analysis

The 3D structures of the SMKIs were prepared and energy optimization was performed by mm2 using Chem3D 18.2. pre‐*let‐7* miRNA was extracted from the reported LIN28–*let‐7* complex (PDB ID: 5UDZ). Blind docking was performed using the HDOCK server. Docking scores were then collected and compared to select the best and least active compounds among the set of 78 SMKIs. The three best‐ranked SMKIs were used for the subsequent MD simulation study.

##### MD Simulation: Model Setup

MD simulations were performed using the Amber20 software package along with AmberTools20. The 3D structures of the SMKIs were prepared, and missing parameters were assigned using the Antechamber module. The parmchk2 utility was used to generate any missing force field parameters based on the General AMBER Force Field (GAFF). Atomic partial charges were calculated using the AM1‐BCC method. The definition of the SMKIs was provided in Table S5‐S7, Supporting Information. H‐atoms were appended by applying the tleap module at the allocated places of the macromolecules. The miRNA structure (pre‐*let*‐*7f*‐*1*) was extracted from the LIN28–*let*‐*7* complex (PDB ID: 5UDZ). The high negative electrostatic potentials around the RNA were neutralized by adding K^+^ ions. 10 Å truncated octahedral shell of pre‐equilibrated TIP3P water was introduced for the explicit solvation.

##### MD Simulation: Forcefields

The leaprc.RNA.OL3 force field was applied to the RNA, while GAFF parameters were used for the small molecules. All parameters were assigned and verified using LEaP from AmberTools.

##### MD Simulation: Simulation Parameters

The energy minimization for the miRNA and the SMKI–miRNA complexes was carried out in two phases. In the first phase, the positional restraints were supplied to conserve the rigidity of the RNA structure by providing the force constants of 500 and 50 kcal mol^–1^, respectively. The following second minimization was executed without any restraint. The minimized structures were heated from 0 to 300 K for 20 ps simulation time at a constant volume with 50 kcal mol^–1^ restraining energy. The whole system was then equilibrated. By including Langevin dynamics, the temperature of the system was controlled with a collision frequency of 1.0 ps^−1^. Subsequent equilibration was performed under constant pressure (NPT) conditions. The equilibration step was extended at 300 K temperature by successional reducing the constraints (50, 40, 30, 20, 10 kcal/mol) for 50 ps per step. Next, the whole system was relaxed with a 1 ns final equilibration run by providing the minimal restraint of only 5 kcal mol^–1^ in ample time.

##### MD Simulation: Production MD

A 1000ns production run was carried out by using the sander module of Amber 20 and the latest force field leaprc.RNA.OL3 obtained from AmberTools 20. A 2 fs time step was used throughout the simulation. The bonds that take account of hydrogen were restricted using the SHAKE algorithm. The required temperature of the system was supported with the help of the Langevin temperature equilibration scheme. The long‐range electrostatics were appraised using periodic boundary conditions, which were developed on the particle mesh Ewald (PME) method.

##### MD Simulation: Post‐Simulation Analyses

Trajectory analyses were performed using the cpptraj module. Structural stability and dynamics were assessed using root–mean‐square deviation (RMSD), root–mean‐square fluctuation (RMSF), and radius of gyration (Rg) calculations. Hydrogen bond occupancy and SASA analyses were performed using cpptraj. Binding free energies of native miRNA and miRNA–SMKI complexes were calculated using the MM/GBSA [molecular mechanics (MM), generalized Born (GB)] approach, implemented in AMBER20. *π*–*π* stacking interactions between the RNA bases and aromatic moieties of the SMKIs were analyzed by monitoring centroid–centroid distances and interplanar angles between aromatic rings using custom scripts in cpptraj and visual inspection in VMD. Molecular visualizations and structural snapshots were generated using PyMOL and VMD.

##### Microscale Thermophoresis

MST measurements were carried out on a Monolith NT.115 system (NanoTemper Technologies) with Cy5‐labeled pre‐*let*‐*7a*−1 (UUA GGG UCA CAC CCA CCA CUG GGA GAU AA, purchased from IDT) and Cy5‐labeled miRNA21 (GUU GAC UGU UGA AUC UCA UGG CAA C, purchased from IDT). Samples were prepared with constant 5 nM RNA and increasing concentration (up to 100–300 µM) of the small molecules in 1 × MST buffer (8 mM Na_2_HPO_4_, 185 mM NaCl, 1 mM EDTA). RNA was prepared by heating at 60 ˚C for 5 min, and it was slowly cooled down to room temperature. In all samples, Tween‐20 was added to a final concentration of 0.05% (v/v). The following parameters were used: 5—20% LED, 20—80% MST power, LaserOn time = 30 s, Laser‐Off time = 5 s. Fluorescence was detected using excitation wavelengths of 600–650 nm and emission wavelengths of 680–685 nm. The resulting data were analyzed by thermophoresis analysis and fitted by the quadratic binding equation in the MST analysis software (NanoTemper Technologies).^[^ ^21^ ^]^

## Supporting Information

Supporting Information for this article is available on the WWW under https://doi.org/CBIC.202500421.

## Conflict of Interest

The authors declare no conflict of interest.

## Supporting information

Supplementary Material

## Data Availability

The data that support the findings of this study are available from the corresponding author upon reasonable request.
