# Supplementary material for: Docking and Molecular Dynamics Simulation‐Based Analysis of Advanced Small‐Molecule Kinase Inhibitors Identified pre‐let‐7 miRNA Binders
Source: Chembiochem. 2025 Aug 26;26(22):e202500421. doi: 10.1002/cbic.202500421 (PMC12631003; doi:10.1002/cbic.202500421)
Supplement: Supplementary file 1 — Supplementary Material [file CBIC-26-e202500421-s001.pdf]

# Supplementary Information

## Docking and Molecular dynamics simulation-based analysis of advanced small-molecule kinase inhibitors identified pre-*let-7* miRNA binders

Soma Roy,[a,b] Yang Liu[a,b,c] and Peng Wu*[a,b,c]

[a] Chemical Genomics Centre, Max Planck Institute of Molecular Physiology, Dortmund 44227, Germany.

[b] Department of Chemical Biology, Max Planck Institute of Molecular Physiology, Dortmund 44227, Germany.

[c] Faculty of Chemistry and Chemical Biology, TU Dortmund University 44227, Germany.

Correspondence: P.W., email: peng.wu@mpi-dortmund.mpg.de

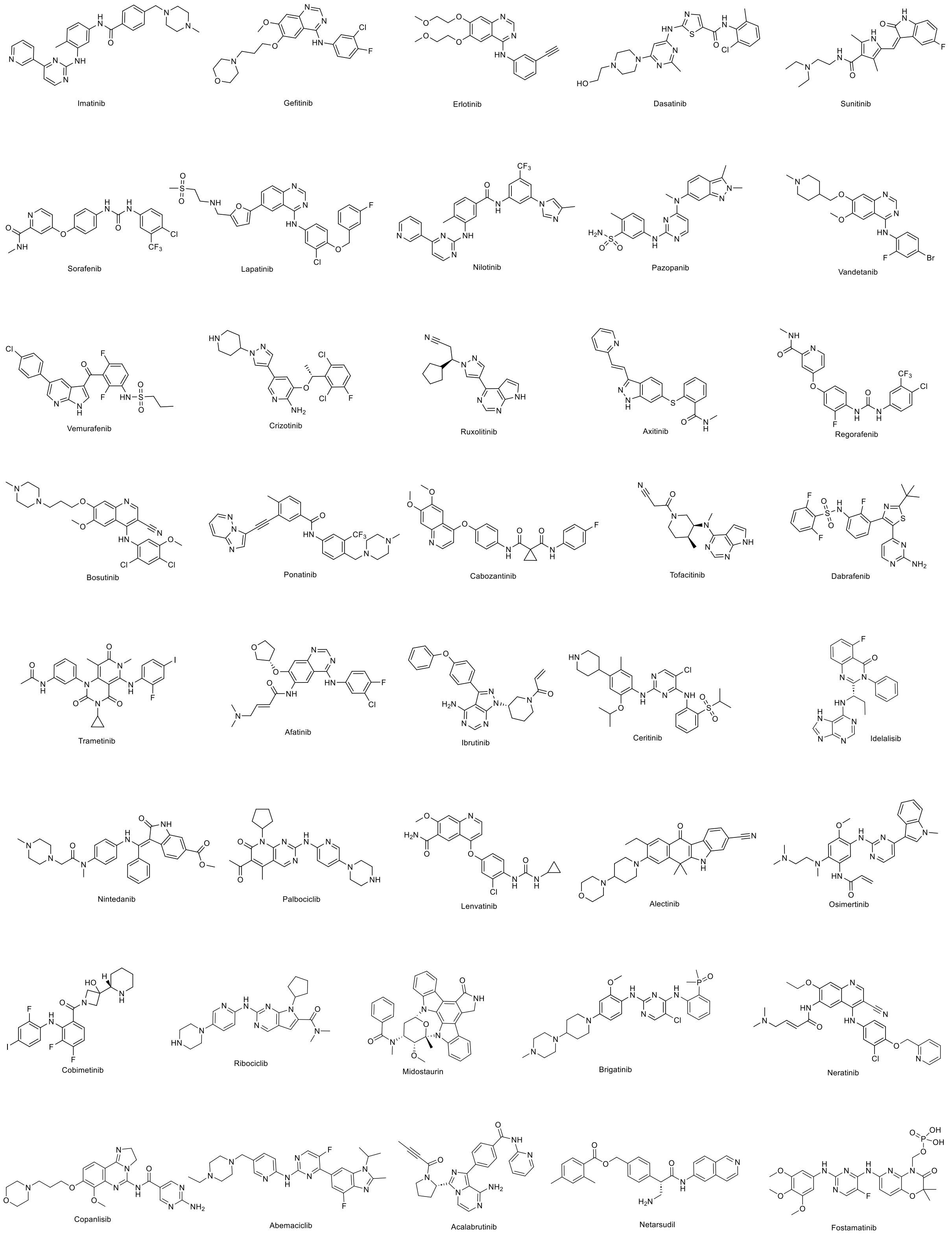


**Figure S1 (Part I).** Structures of the 78 FDA-approved small-molecule kinase inhibitors (SMKIs) evaluated in this study.

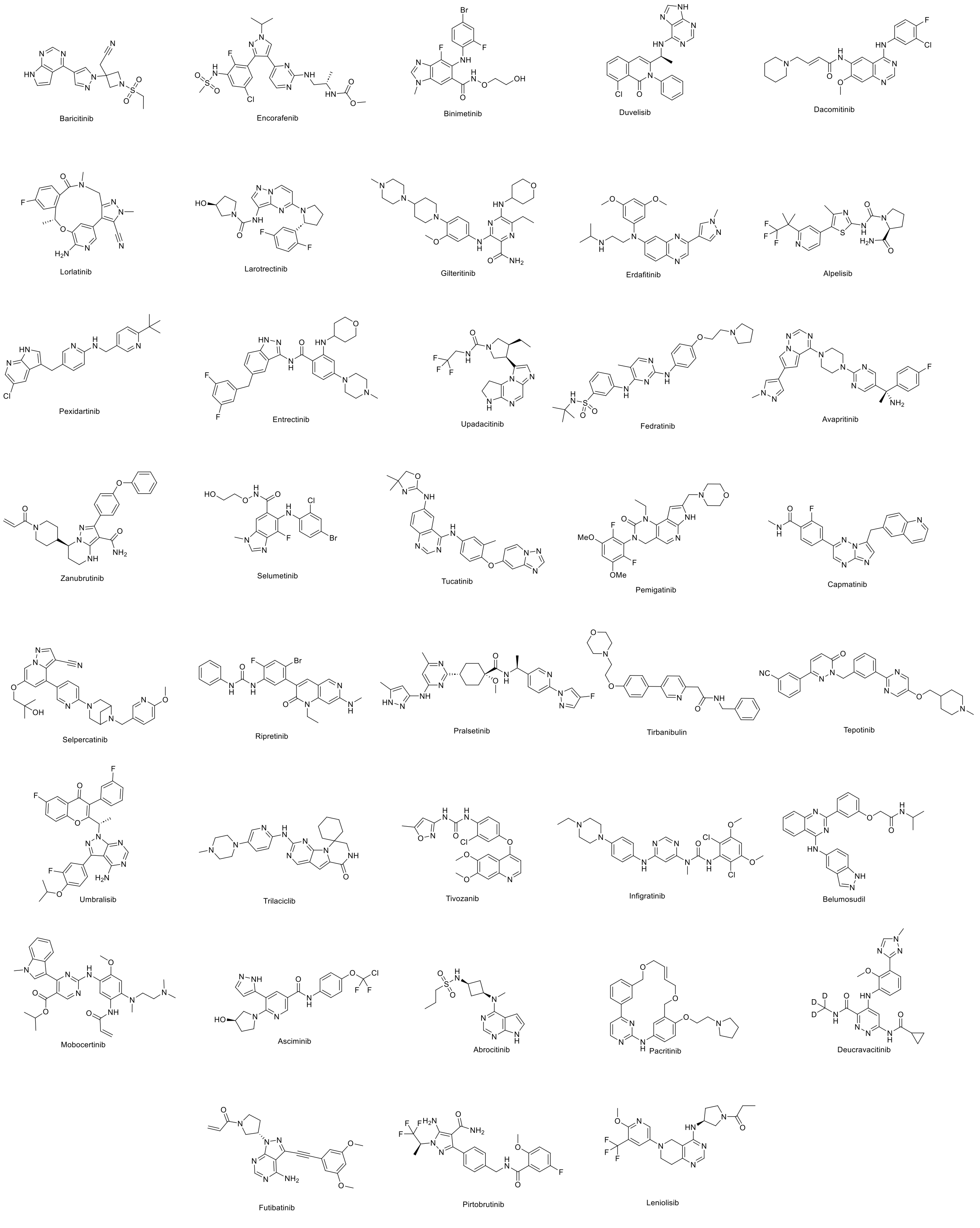


**Figure S1 (Part II).** Structures of the 78 FDA-approved small-molecule kinase inhibitors (SMKIs) evaluated in this study.

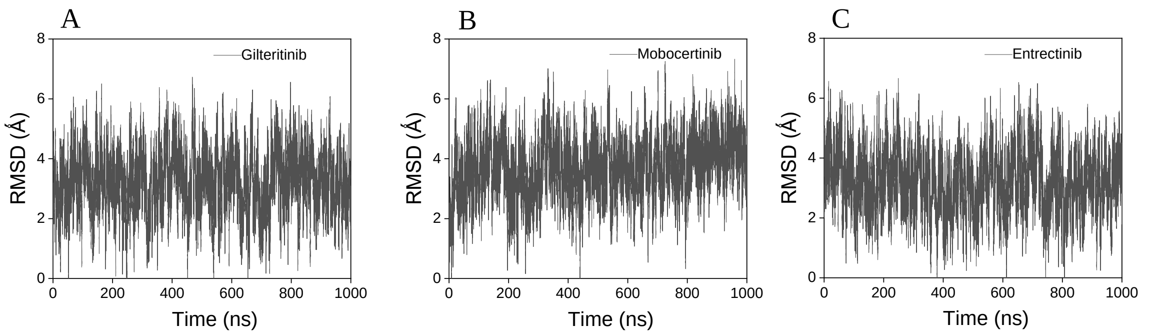


**Fig S2.** RMSD of the ligands throughout the simulation period (A) Gilteritinib, (B) Mobocertinib, (C) Entrectinib.

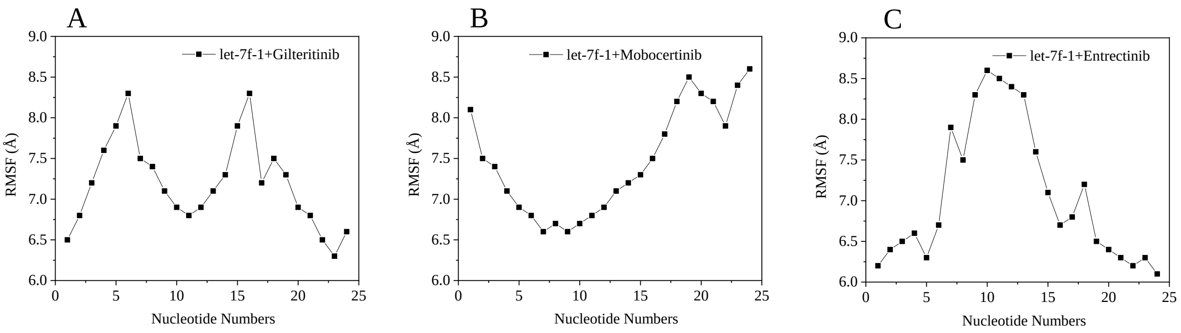


**Fig S3.** A plot of RMSF of the individual residue of pre-*let-7* and SMKI–pre-*let-7* complexes with gilteritinib (A), mobocertinib (B), and entrectinib (C).

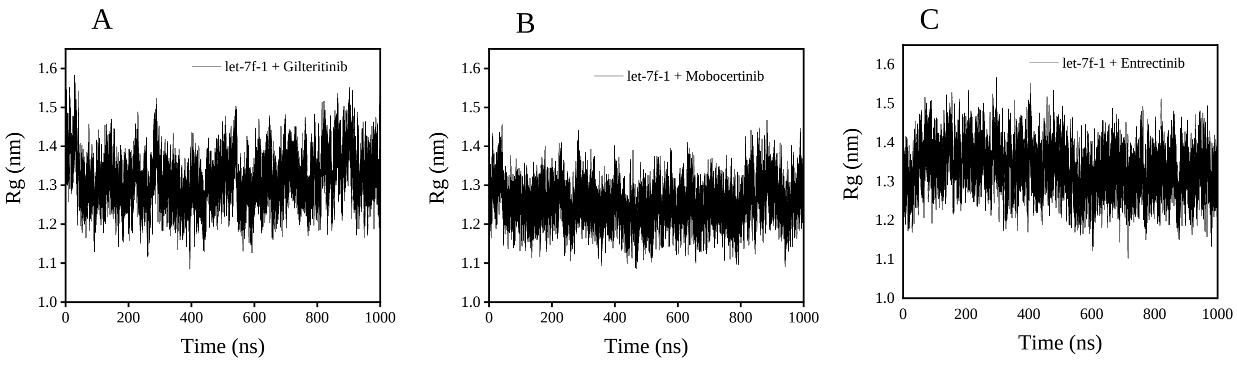


**Fig S4.** The radius of gyration (Rg) values of pre-*let-7* and SMKI–pre-*let-7* complexes with (A) gilteritinib, (B) mobocertinib, (C) entrectinib during 1000 ns of production runs.

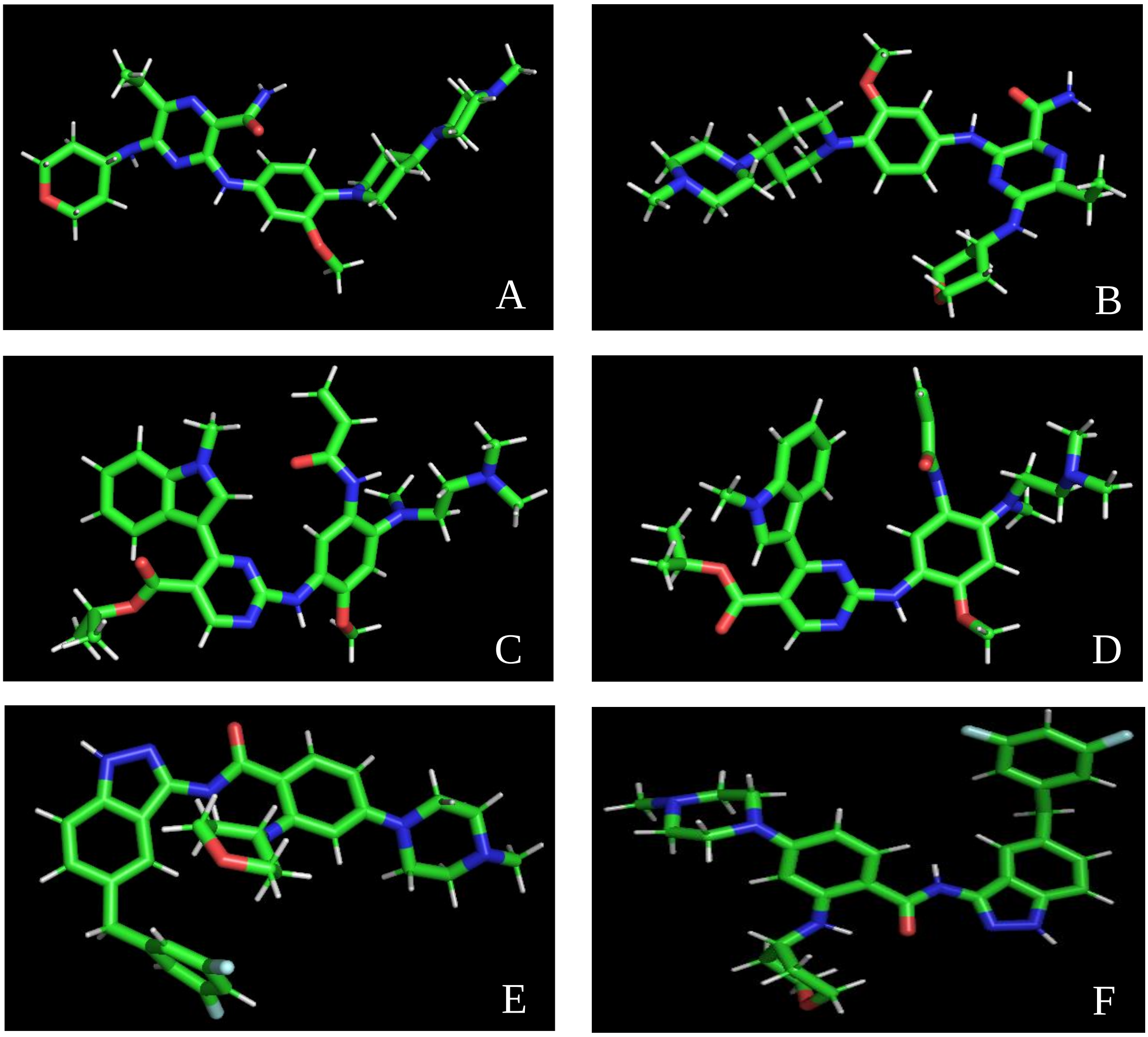


**Fig S5.** Low energy conformers of the SMKIs. Gilteritinib (A) in water, (B) in complex; Mobocertinib (C) in water, (D) in complex; Entrectinib (E) in water, (F) in complex.

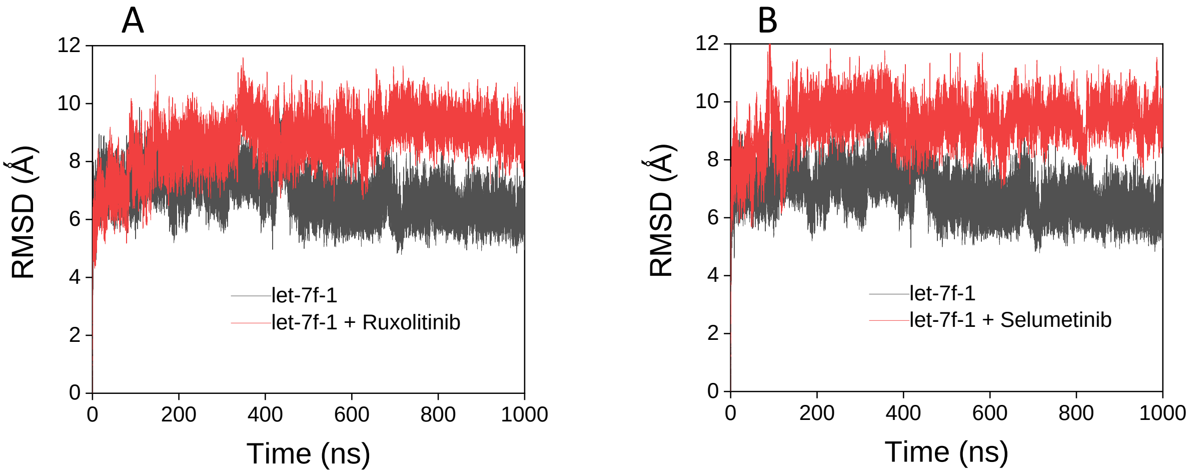


C

| Entry | $E_{VDW}$ | $E_{ELEC}$ | $E_{GB}$ | $E_{SURF}$ | $\Delta G_{GAS}$ | $\Delta G_{SOLV}$ | $\Delta G_{TOTAL}$ |
|---|---|---|---|---|---|---|---|
| Ruxolitinib–miRNA | -39.1 ± 0.1 | 10.1 ± 0.2 | 20.5 ± 0.5 | -2.4 ± 0.01 | -29.0 ± 0.2 | 18.1 ± 0.5 | -10.9 ± 0.1 |
| Selumetinib–miRNA | -30.5 ± 0.4 | 10.9 ± 0.2 | 15.3 ± 0.5 | -5.1 ± 0.02 | -19.6 ± 0.5 | 10.2 ± 0.3 | -9.4 ± 0.2 |

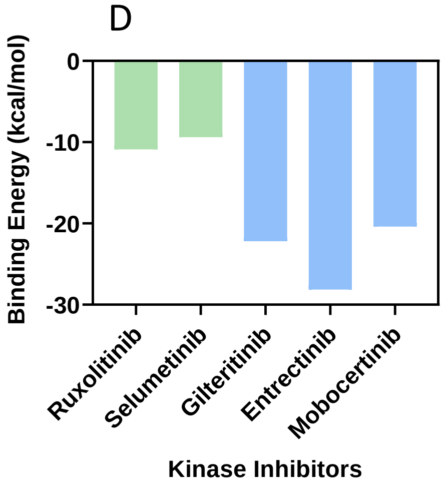


**Figure S6.** MD simulation plots. (A-B) SMKI–pre-*let*-7 complexes with ruxolitinib, and selumetinib, in the duration of 1000 ns. (C) Contribution to the binding free energies (kcal mol$^{-1}$) acquired from the MM/GBSA calculation for the ruxolitinib–miRNA and selumetinib–RNA complexes. (D) Comparative binding energies obtained from MM/GBSA calculation.

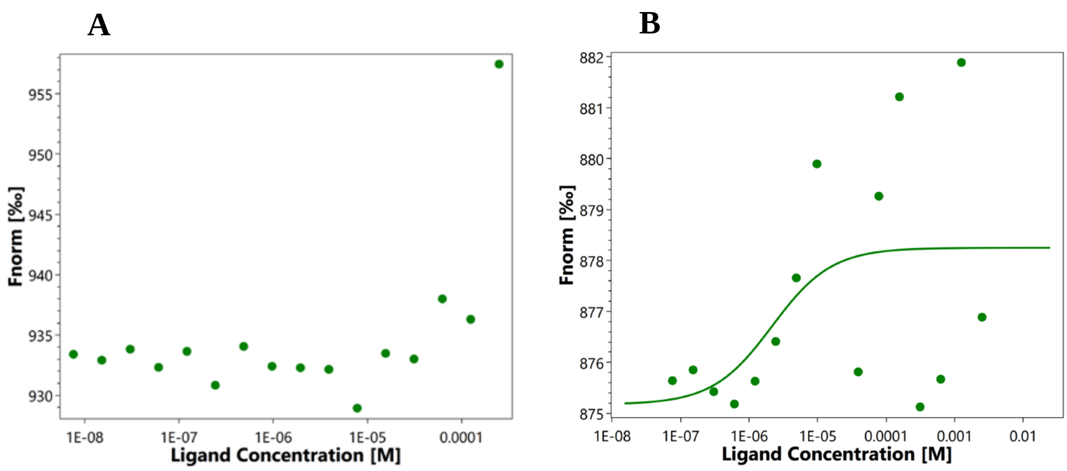


**Figure S7.** Thermophoresis analysis of the SMKIs with miRNA21. (A) gilteritinib, (B) entrectinib.

**Table S1.** Docking scores of the small-molecule kinase inhibitors with pre-*let-7* miRNA.

| No. | Compounds | Docking Score |
|---|---|---|
| 1 | Imatinib | -136.94 |
| 2 | Gefitinib | -138.54 |
| 3 | Erlotinib | -141.44 |
| 4 | Dasatinib | -146.42 |
| 5 | Sunitinib | -127.75 |
| 6 | Sorafenib | -143.52 |
| 7 | Lapatinib | -155.60 |
| 8 | Nilotinib | -150.91 |
| 9 | Pazopanib | -143.79 |
| 10 | Vandetanib | -134.80 |
| 11 | Vemurafenib | -133.16 |
| 12 | Crizotinib | -138.26 |
| 13 | Ruxolitinib | -122.48 |
| 14 | Auxitinib | -141.28 |
| 15 | Regorafenib | -140.83 |
| 16 | Bosutinib | -143.15 |
| 17 | Ponatinib | -152.13 |
| 18 | Cabozantinib | -143.48 |
| 19 | Tofacitinib | -133.21 |
| 20 | Dabrafenib | -138.88 |
| 21 | Trametinib | -144.60 |
| 22 | Afatinib | -148.32 |
| 23 | Ibrutinib | -154.23 |
| 24 | Ceritinib | -139.58 |
| 25 | Idelalisib | -160.97 |
| 26 | Nintedanib | -143.46 |
| 27 | Palbociclib | -169.92 |
| 28 | Lenvatinib | -132.02 |
| 29 | Alectinib | -133.74 |

| | | |
|---|---|---|
| 30 | Osimertinib | -153.46 |
| 31 | Cobimetinib | -117.81 |
| 32 | Ribociclib | -151.16 |
| 33 | Midostaurin | -153.65 |
| 34 | Brigatinib | -151.47 |
| 35 | Neratinib | -150.97 |
| 36 | Copanlisib | -167.10 |
| 37 | Abemaciclib | -151.47 |
| 38 | Acalabrutinib | -159.89 |
| 39 | Netarsudil | -125.29 |
| 40 | Fostamatinib | -166.44 |
| 41 | Baricitinib | -137.98 |
| 42 | Encorafenib | -147.97 |
| 43 | Binimetinib | -129.87 |
| 44 | Duvelisib | -149.30 |
| 45 | Dacomitinib | -129.39 |
| 46 | Lorlatinib | -156.01 |
| 47 | Larotrectinib | -153.09 |
| 48 | Gilteritinib | -183.16 |
| 49 | Erdafitinib | -133.35 |
| 50 | Alpelisib | -137.43 |
| 51 | Pexidartinib | -142.24 |
| 52 | Entrectinib | -177.75 |
| 53 | Upadacitinib | -135.24 |
| 54 | Fedratinib | -134.92 |
| 55 | Avapritinib | -156.53 |
| 56 | Zanubrutinib | -139.58 |
| 57 | Selumetinib | -126.95 |
| 58 | Tucatinib | -167.78 |
| 59 | Pemigatinib | -152.22 |
| 60 | Capmatinib | -144.89 |
| 61 | Selpercatinib | -159.20 |

| | | |
|---|---|---|
| 62 | Ripretinib | -131.65 |
| 63 | Pralsetinib | -152.45 |
| 64 | Tirbanibulin | -115.92 |
| 65 | Tepotinib | -144.81 |
| 66 | Umbralisib | -157.50 |
| 67 | Trilaciclib | -156.15 |
| 68 | Tivozanib | -146.07 |
| 69 | Infigratinib | -149.66 |
| 70 | Belumosudil | -151.12 |
| 71 | Mobocertinib | -179.90 |
| 72 | Asciminib | -144.54 |
| 73 | Abrocitinib | -134.07 |
| 74 | Pacritinib | -142.93 |
| 75 | Deucravacitinib | -160.29 |
| 76 | Futibatinib | -147.90 |
| 77 | Pirtobrutinib | -141.29 |
| 78 | Leniolisib | -131.64 |

**Table S2.** Hydrogen bond occurrence of the ligand-RNA complexes during the final molecular dynamics simulation for 1000 ns time period.

| Entry | H-bonding interactions | Distance (Å) |
|---|---|---|
| pre-*let*-7 + Gilteritinib | O3'_G4@LIG_N3_96 | 3.1 |
| | O2'_U14@LIG_O4_67 | 2.9 |
| pre-*let*-7 + Mobocertinib | OP1_A15@LIG_H7_34 | 3.0 |
| pre-*let*-7 + Entrectinib | OP2_A10@LIG_H6_29 | 2.8 |
| | O1_U11@LIG_N3_83 | 2.9 |
| | H1_U13@LIG_O2_37 | 3.0 |

**Table S3.** π-π interaction energies (kcal $mol^{-1}$) of the ligand-RNA complexes.

| Entry | π-π interaction energy (kcal $mol^{-1}$) | | | |
|---|---|---|---|---|
| | **200 ns** | **400 ns** | **800 ns** | **1000 ns** |
| pre-*let*-7 + Gilteritinib | -1.5 | -1.6 | -1.7 | -1.7 |
| pre-*let*-7 + Mobocertinib | -1.3 | -1.4 | -1.8 | -1.9 |
| pre-*let*-7 + Entrectinib | -1.8 | -1.6 | -1.9 | -2.1 |

**Table S4.** Strain energy calculations of the SMKIs.

| SMKI | Single-point energy at the bound state (hartree) | Single-point energy at the unbound state (hartree) | Strain energy (hartree) | Strain energy (kcal/mol) |
|---|---|---|---|---|
| Gilteritinib | -2456.45902 | -2456.46316 | 0.00414 | 2.6 |
| Mobocertinib | -2383.80365 | -2383.81050 | 0.00685 | 4.3 |
| Entrectinib | -2445.43821 | -2445.44267 | 0.00446 | 2.8 |

## Ligand Strain Energy Calculation

Ligand strain energy (ΔE) is defined as the energy difference between the ligand's conformation in the bound complex and its unbound state in solvent (water).[1]

$\Delta E = E_{bound} - E_{unbound}$

The lowest-energy conformers (global minima) of the ligands were extracted from both the bound and water-solvated simulations using the *cpptraj* utility in AMBER 20. Single-point energy calculations for these global minima conformers were then performed using density functional theory (DFT) with the Minnesota 06 (M06)[2] functional and the 6-311+G(d,p) basis set.[3] Finally, strain potential energies were determined based on the energy difference between the bound and unbound ligand states, following the established procedure.[3]

**Table S5.** Definition of the ligand gilteritinib (in Mol2 format) including all of the charges and atom types to load into Leap.

```
@<TRIPOS>MOLECULE
UNK
   84    88     1     0     0
SMALL
bcc


@<TRIPOS>ATOM
      1 N          41.8850     6.1070    22.4740 nb         1 UNK      -0.714000
      2 C          43.1550     6.1730    22.4460 ca         1 UNK       0.505900
      3 C1         43.8320     5.0160    22.6310 ca         1 UNK       0.257500
      4 N1         43.1960     3.9200    22.6460 nb         1 UNK      -0.593000
      5 C2         41.9320     3.8790    22.6080 ca         1 UNK       0.318300
      6 C3         41.2250     5.0250    22.5460 ca         1 UNK       0.505900
      7 N2         39.9630     5.2050    22.5260 nh         1 UNK      -0.797900
      8 C4         38.8190     4.2710    22.5730 c3         1 UNK       0.177500
      9 C5         41.4250     2.4540    22.6790 c3         1 UNK      -0.117700
     10 C6         41.2770     1.8490    21.2760 c3         1 UNK      -0.091100
     11 N3         43.5580     7.3620    22.2160 nh         1 UNK      -0.689300
     12 C7         44.5880     8.0100    21.8320 ca         1 UNK       0.110600
     13 C8         45.1710     4.8990    22.8700 c          1 UNK       0.592600
     14 N4         45.7640     6.0010    23.3960 n          1 UNK      -0.638000
     15 O          45.8340     3.8900    22.8110 o          1 UNK      -0.600100
     16 C9         44.5260     9.3440    21.8980 ca         1 UNK      -0.180000
     17 C10        45.5760    10.0910    21.5330 ca         1 UNK      -0.134000
     18 C11        46.7410     9.6130    21.0360 ca         1 UNK       0.089600
     19 C12        46.7890     8.2530    20.9140 ca         1 UNK       0.143100
     20 C13        45.7180     7.5240    21.3020 ca         1 UNK      -0.217000
     21 N5         47.6860    10.4330    20.7280 nh         1 UNK      -0.612000
```

| | | | | | | | | |
|---|---|---|---|---|---|---|---|---|
| 22 | C14 | 49.0200 | 10.0490 | 20.1800 | c3 | 1 | UNK | 0.180800 |
| 23 | C15 | 49.9830 | 10.1230 | 21.3750 | c3 | 1 | UNK | -0.126400 |
| 24 | C16 | 50.0080 | 11.5580 | 21.9580 | c3 | 1 | UNK | 0.189500 |
| 25 | C17 | 48.5790 | 12.0810 | 22.2450 | c3 | 1 | UNK | -0.126400 |
| 26 | C18 | 47.6530 | 11.9020 | 21.0250 | c3 | 1 | UNK | 0.180800 |
| 27 | N6 | 50.9650 | 11.6770 | 23.0620 | n3 | 1 | UNK | -0.733600 |
| 28 | C19 | 51.2370 | 13.0640 | 23.4210 | c3 | 1 | UNK | 0.171800 |
| 29 | C20 | 52.3590 | 13.1500 | 24.4690 | c3 | 1 | UNK | 0.167800 |
| 30 | N7 | 51.9990 | 12.4120 | 25.6700 | n3 | 1 | UNK | -0.732600 |
| 31 | C21 | 51.7110 | 11.0300 | 25.3240 | c3 | 1 | UNK | 0.167800 |
| 32 | C22 | 50.5930 | 10.9480 | 24.2680 | c3 | 1 | UNK | 0.171800 |
| 33 | C23 | 53.0450 | 12.4980 | 26.6780 | c3 | 1 | UNK | 0.157100 |
| 34 | C24 | 38.3590 | 4.0210 | 24.0160 | c3 | 1 | UNK | -0.127400 |
| 35 | C25 | 37.0980 | 3.1600 | 24.0190 | c3 | 1 | UNK | 0.126900 |
| 36 | O1 | 36.0670 | 3.7850 | 23.2970 | os | 1 | UNK | -0.412600 |
| 37 | C26 | 36.4060 | 3.9730 | 21.9450 | c3 | 1 | UNK | 0.126900 |
| 38 | C27 | 37.6290 | 4.8770 | 21.8130 | c3 | 1 | UNK | -0.127400 |
| 39 | O2 | 47.9100 | 7.6090 | 20.4440 | os | 1 | UNK | -0.321900 |
| 40 | C28 | 48.0720 | 6.2150 | 20.5760 | c3 | 1 | UNK | 0.103700 |
| 41 | H | 39.6990 | 6.1520 | 22.4680 | hn | 1 | UNK | 0.391800 |
| 42 | H1 | 39.1390 | 3.3500 | 22.1320 | h1 | 1 | UNK | 0.061700 |
| 43 | H2 | 42.1180 | 1.8640 | 23.2420 | hc | 1 | UNK | 0.061200 |
| 44 | H3 | 40.4640 | 2.4580 | 23.1500 | hc | 1 | UNK | 0.061200 |
| 45 | H4 | 42.2300 | 1.8370 | 20.7900 | hc | 1 | UNK | 0.043700 |
| 46 | H5 | 40.5920 | 2.4390 | 20.7030 | hc | 1 | UNK | 0.043700 |
| 47 | H6 | 40.9050 | 0.8490 | 21.3560 | hc | 1 | UNK | 0.043700 |
| 48 | H7 | 42.8250 | 7.9940 | 22.3960 | hn | 1 | UNK | 0.399800 |
| 49 | H8 | 45.2620 | 6.8450 | 23.4640 | hn | 1 | UNK | 0.313500 |
| 50 | H9 | 46.6960 | 5.9560 | 23.7100 | hn | 1 | UNK | 0.313500 |
| 51 | H10 | 43.6630 | 9.8010 | 22.2320 | ha | 1 | UNK | 0.131000 |

| | | | | | | | |
|---|---|---|---|---|---|---|---|
| 52 | H11 | 45.4900 | 11.1140 | 21.6390 | ha | 1 UNK | 0.136000 |
| 53 | H12 | 45.7770 | 6.5010 | 21.1790 | ha | 1 UNK | 0.162000 |
| 54 | H13 | 49.3210 | 10.7310 | 19.4130 | h1 | 1 UNK | 0.059450 |
| 55 | H14 | 49.0050 | 9.0750 | 19.7380 | h1 | 1 UNK | 0.059450 |
| 56 | H15 | 50.9680 | 9.8560 | 21.0520 | hc | 1 UNK | 0.051700 |
| 57 | H16 | 49.6490 | 9.4450 | 22.1330 | hc | 1 UNK | 0.051700 |
| 58 | H17 | 50.3860 | 12.2290 | 21.2150 | h1 | 1 UNK | 0.055700 |
| 59 | H18 | 48.1710 | 11.5400 | 23.0730 | hc | 1 UNK | 0.051700 |
| 60 | H19 | 48.6430 | 13.1260 | 22.4660 | hc | 1 UNK | 0.051700 |
| 61 | H20 | 46.6570 | 12.2170 | 21.2590 | h1 | 1 UNK | 0.059450 |
| 62 | H21 | 47.9700 | 12.4890 | 20.1890 | h1 | 1 UNK | 0.059450 |
| 63 | H22 | 50.3480 | 13.5030 | 23.8230 | h1 | 1 UNK | 0.036450 |
| 64 | H23 | 51.5490 | 13.5890 | 22.5430 | h1 | 1 UNK | 0.036450 |
| 65 | H24 | 52.5240 | 14.1760 | 24.7240 | h1 | 1 UNK | 0.035700 |
| 66 | H25 | 53.2490 | 12.7220 | 24.0560 | h1 | 1 UNK | 0.035700 |
| 67 | H26 | 52.5970 | 10.5750 | 24.9320 | h1 | 1 UNK | 0.035700 |
| 68 | H27 | 51.3860 | 10.5180 | 26.2060 | h1 | 1 UNK | 0.035700 |
| 69 | H28 | 50.4210 | 9.9220 | 24.0180 | h1 | 1 UNK | 0.036450 |
| 70 | H29 | 49.7050 | 11.3870 | 24.6740 | h1 | 1 UNK | 0.036450 |
| 71 | H30 | 53.2330 | 13.5250 | 26.9100 | h1 | 1 UNK | 0.031367 |
| 72 | H31 | 53.9400 | 12.0480 | 26.3030 | h1 | 1 UNK | 0.031367 |
| 73 | H32 | 52.7310 | 11.9840 | 27.5620 | h1 | 1 UNK | 0.031367 |
| 74 | H33 | 38.1500 | 4.9580 | 24.4890 | hc | 1 UNK | 0.060450 |
| 75 | H34 | 39.1340 | 3.5110 | 24.5490 | hc | 1 UNK | 0.060450 |
| 76 | H35 | 37.3180 | 2.2140 | 23.5700 | h1 | 1 UNK | 0.050200 |
| 77 | H36 | 36.7760 | 3.0250 | 25.0310 | h1 | 1 UNK | 0.050200 |
| 78 | H37 | 36.6230 | 3.0230 | 21.5020 | h1 | 1 UNK | 0.050200 |
| 79 | H38 | 35.5810 | 4.4370 | 21.4460 | h1 | 1 UNK | 0.050200 |
| 80 | H39 | 37.4030 | 5.8400 | 22.2200 | hc | 1 UNK | 0.060450 |
| 81 | H40 | 37.8850 | 4.9700 | 20.7780 | hc | 1 UNK | 0.060450 |

| | | | | | | | | |
|---|---|---|---|---|---|---|---|---|
| 82 | H41 | 49.0070 | 5.9220 | 20.1460 | h1 | 1 | UNK | 0.053033 |
| 83 | H42 | 47.2740 | 5.7140 | 20.0690 | h1 | 1 | UNK | 0.053033 |
| 84 | H43 | 48.0580 | 5.9510 | 21.6130 | h1 | 1 | UNK | 0.053033 |

@<TRIPOS>BOND

| | | | |
|---|---|---|---|
| 1 | 1 | 2 | ar |
| 2 | 1 | 6 | ar |
| 3 | 2 | 3 | ar |
| 4 | 2 | 11 | 1 |
| 5 | 3 | 4 | ar |
| 6 | 3 | 13 | 1 |
| 7 | 4 | 5 | ar |
| 8 | 5 | 6 | ar |
| 9 | 5 | 9 | 1 |
| 10 | 6 | 7 | 1 |
| 11 | 7 | 8 | 1 |
| 12 | 7 | 41 | 1 |
| 13 | 8 | 34 | 1 |
| 14 | 8 | 38 | 1 |
| 15 | 8 | 42 | 1 |
| 16 | 9 | 10 | 1 |
| 17 | 9 | 43 | 1 |
| 18 | 9 | 44 | 1 |
| 19 | 10 | 45 | 1 |
| 20 | 10 | 46 | 1 |
| 21 | 10 | 47 | 1 |
| 22 | 11 | 12 | 1 |
| 23 | 11 | 48 | 1 |
| 24 | 12 | 16 | ar |
| 25 | 12 | 20 | ar |
| 26 | 13 | 14 | 1 |

27 13 15 2
28 14 49 1
29 14 50 1
30 16 17 ar
31 16 51 1
32 17 18 ar
33 17 52 1
34 18 19 ar
35 18 21 1
36 19 20 ar
37 19 39 1
38 20 53 1
39 21 22 1
40 21 26 1
41 22 23 1
42 22 54 1
43 22 55 1
44 23 24 1
45 23 56 1
46 23 57 1
47 24 25 1
48 24 27 1
49 24 58 1
50 25 26 1
51 25 59 1
52 25 60 1
53 26 61 1
54 26 62 1
55 27 28 1
56 27 32 1

| | | | |
|---|---|---|---|
| 57 | 28 | 29 | 1 |
| 58 | 28 | 63 | 1 |
| 59 | 28 | 64 | 1 |
| 60 | 29 | 30 | 1 |
| 61 | 29 | 65 | 1 |
| 62 | 29 | 66 | 1 |
| 63 | 30 | 31 | 1 |
| 64 | 30 | 33 | 1 |
| 65 | 31 | 32 | 1 |
| 66 | 31 | 67 | 1 |
| 67 | 31 | 68 | 1 |
| 68 | 32 | 69 | 1 |
| 69 | 32 | 70 | 1 |
| 70 | 33 | 71 | 1 |
| 71 | 33 | 72 | 1 |
| 72 | 33 | 73 | 1 |
| 73 | 34 | 35 | 1 |
| 74 | 34 | 74 | 1 |
| 75 | 34 | 75 | 1 |
| 76 | 35 | 36 | 1 |
| 77 | 35 | 76 | 1 |
| 78 | 35 | 77 | 1 |
| 79 | 36 | 37 | 1 |
| 80 | 37 | 38 | 1 |
| 81 | 37 | 78 | 1 |
| 82 | 37 | 79 | 1 |
| 83 | 38 | 80 | 1 |
| 84 | 38 | 81 | 1 |
| 85 | 39 | 40 | 1 |
| 86 | 40 | 82 | 1 |

87 40 83 1

88 40 84 1

@<TRIPOS>SUBSTRUCTURE

1 UNK 1 TEMP 0 **** **** 0 ROOT

**Table S6.** Definition of the ligand Mobocertinib (in Mol2 format) including all of the charges and atom types to load into Leap.

```
@<TRIPOS>MOLECULE
LIG
   82    85     1     0     0
SMALL
bcc


@<TRIPOS>ATOM
      1 N          49.0600     3.8910    33.5000 nh         1 LIG      -0.683300
      2 H          48.6310     3.3330    32.7160 hn         1 LIG       0.449800
      3 C          50.3310     3.7900    33.5110 ca         1 LIG       0.882900
      4 N1         50.9520     4.8860    33.6490 nb         1 LIG      -0.725000
      5 C1         52.2130     4.8810    33.6910 ca         1 LIG       0.501200
      6 C2         52.9690     3.7680    33.5950 ca         1 LIG      -0.469200
      7 C3         52.2600     2.6160    33.4800 ca         1 LIG       0.628100
      8 N2         50.9940     2.7160    33.4050 nb         1 LIG      -0.750000
      9 C4         54.3350     3.9110    33.5340 c          1 LIG       0.673700
     10 O          55.0330     3.4500    34.4200 o          1 LIG      -0.514000
     11 O1         54.9560     4.6510    32.5690 os         1 LIG      -0.415900
     12 C5         55.0170     4.2160    31.2420 c3         1 LIG       0.144100
     13 C6         56.3840     3.6040    30.9300 c3         1 LIG      -0.109100
     14 C7         54.7550     5.4350    30.3530 c3         1 LIG      -0.109100
     15 C8         52.6860     1.3320    33.4290 cc         1 LIG      -0.266000
     16 C9         52.5770     0.3920    34.3890 cd         1 LIG      -0.046100
     17 N3         53.0270    -0.7020    33.9360 na         1 LIG      -0.065400
     18 C10        53.0400    -1.9520    34.7110 c3         1 LIG       0.026600
     19 C11        53.4420    -0.6190    32.7440 ca         1 LIG      -0.058200
     20 C12        53.9770    -1.5290    31.9200 ca         1 LIG      -0.139000
     21 C13        53.2360     0.6610    32.3880 ca         1 LIG      -0.049800
```

| | | | | | | | | |
|---|---|---|---|---|---|---|---|---|
| 22 | C14 | 54.3470 | -1.1280 | 30.6940 | ca | 1 | LIG | -0.118000 |
| 23 | C15 | 54.1970 | 0.1560 | 30.3320 | ca | 1 | LIG | -0.154000 |
| 24 | C16 | 53.6530 | 1.0530 | 31.1720 | ca | 1 | LIG | -0.077000 |
| 25 | C17 | 48.1600 | 4.4890 | 34.1760 | ca | 1 | LIG | 0.114600 |
| 26 | C18 | 47.9510 | 4.0010 | 35.4130 | ca | 1 | LIG | 0.078100 |
| 27 | C19 | 47.0280 | 4.6430 | 36.1560 | ca | 1 | LIG | -0.158000 |
| 28 | C20 | 46.2550 | 5.6980 | 35.7990 | ca | 1 | LIG | 0.092600 |
| 29 | C21 | 46.4620 | 6.1210 | 34.5200 | ca | 1 | LIG | 0.030600 |
| 30 | C22 | 47.4070 | 5.5180 | 33.7700 | ca | 1 | LIG | -0.092000 |
| 31 | O2 | 48.7200 | 2.9410 | 35.8480 | os | 1 | LIG | -0.340900 |
| 32 | C23 | 49.3900 | 3.0150 | 37.0860 | c3 | 1 | LIG | 0.118700 |
| 33 | N4 | 45.4160 | 6.1790 | 36.6530 | nh | 1 | LIG | -0.668000 |
| 34 | C24 | 45.7770 | 7.2150 | 37.6580 | c3 | 1 | LIG | 0.181100 |
| 35 | C25 | 43.9880 | 5.7120 | 36.6940 | c3 | 1 | LIG | 0.184800 |
| 36 | C26 | 42.9890 | 6.8560 | 36.4330 | c3 | 1 | LIG | 0.133800 |
| 37 | N5 | 41.6950 | 6.4020 | 35.9310 | n3 | 1 | LIG | -0.742600 |
| 38 | C27 | 41.4330 | 5.0360 | 36.3610 | c3 | 1 | LIG | 0.152600 |
| 39 | C28 | 40.6050 | 7.2760 | 36.3420 | c3 | 1 | LIG | 0.152600 |
| 40 | N6 | 45.7830 | 7.1470 | 33.9600 | n | 1 | LIG | -0.479100 |
| 41 | C29 | 45.8260 | 8.5130 | 34.0480 | c | 1 | LIG | 0.662300 |
| 42 | O3 | 46.6910 | 9.1370 | 34.6130 | o | 1 | LIG | -0.591100 |
| 43 | H1 | 45.1370 | 6.7980 | 33.2840 | hn | 1 | LIG | 0.328500 |
| 44 | C30 | 44.7870 | 9.0850 | 33.3840 | ce | 1 | LIG | -0.243200 |
| 45 | C31 | 44.2540 | 10.2700 | 33.7220 | c2 | 1 | LIG | -0.132000 |
| 46 | H2 | 52.6970 | 5.7850 | 33.8060 | h4 | 1 | LIG | 0.039100 |
| 47 | H3 | 54.2860 | 3.4550 | 31.0650 | h1 | 1 | LIG | 0.060700 |
| 48 | H4 | 57.1480 | 4.3350 | 31.0950 | hc | 1 | LIG | 0.048367 |
| 49 | H5 | 56.4080 | 3.2870 | 29.9080 | hc | 1 | LIG | 0.048367 |
| 50 | H6 | 56.5520 | 2.7620 | 31.5690 | hc | 1 | LIG | 0.048367 |
| 51 | H7 | 55.4960 | 6.1830 | 30.5460 | hc | 1 | LIG | 0.048367 |

| | | | | | | | | |
|---|---|---|---|---|---|---|---|---|
| 52 | H8 | 53.7840 | 5.8310 | 30.5670 | hc | 1 | LIG | 0.048367 |
| 53 | H9 | 54.8020 | 5.1430 | 29.3250 | hc | 1 | LIG | 0.048367 |
| 54 | H10 | 52.1910 | 0.5390 | 35.3350 | h4 | 1 | LIG | 0.198000 |
| 55 | H11 | 53.4660 | -2.7350 | 34.1190 | h1 | 1 | LIG | 0.054700 |
| 56 | H12 | 52.0390 | -2.2140 | 34.9820 | h1 | 1 | LIG | 0.054700 |
| 57 | H13 | 53.6250 | -1.8160 | 35.5970 | h1 | 1 | LIG | 0.054700 |
| 58 | H14 | 54.1010 | -2.5090 | 32.2190 | ha | 1 | LIG | 0.132000 |
| 59 | H15 | 54.7470 | -1.8080 | 30.0290 | ha | 1 | LIG | 0.129000 |
| 60 | H16 | 54.5040 | 0.4590 | 29.3940 | ha | 1 | LIG | 0.130000 |
| 61 | H17 | 53.5560 | 2.0390 | 30.8840 | ha | 1 | LIG | 0.151000 |
| 62 | H18 | 46.8930 | 4.2880 | 37.1150 | ha | 1 | LIG | 0.153000 |
| 63 | H19 | 47.5600 | 5.8760 | 32.8140 | ha | 1 | LIG | 0.185000 |
| 64 | H20 | 49.9310 | 2.1070 | 37.2530 | h1 | 1 | LIG | 0.046367 |
| 65 | H21 | 48.6760 | 3.1540 | 37.8700 | h1 | 1 | LIG | 0.046367 |
| 66 | H22 | 50.0720 | 3.8400 | 37.0740 | h1 | 1 | LIG | 0.046367 |
| 67 | H23 | 46.8080 | 7.4770 | 37.5450 | h1 | 1 | LIG | 0.035700 |
| 68 | H24 | 45.6120 | 6.8300 | 38.6420 | h1 | 1 | LIG | 0.035700 |
| 69 | H25 | 45.1700 | 8.0830 | 37.5090 | h1 | 1 | LIG | 0.035700 |
| 70 | H26 | 43.8510 | 4.9590 | 35.9460 | h1 | 1 | LIG | 0.054700 |
| 71 | H27 | 43.7980 | 5.3290 | 37.6750 | h1 | 1 | LIG | 0.054700 |
| 72 | H28 | 42.8300 | 7.3810 | 37.3510 | h1 | 1 | LIG | 0.036700 |
| 73 | H29 | 43.4170 | 7.4790 | 35.6760 | h1 | 1 | LIG | 0.036700 |
| 74 | H30 | 40.4820 | 4.7200 | 35.9850 | h1 | 1 | LIG | 0.033867 |
| 75 | H31 | 41.4260 | 4.9940 | 37.4300 | h1 | 1 | LIG | 0.033867 |
| 76 | H32 | 42.1980 | 4.3900 | 35.9840 | h1 | 1 | LIG | 0.033867 |
| 77 | H33 | 39.6820 | 6.9030 | 35.9510 | h1 | 1 | LIG | 0.033867 |
| 78 | H34 | 40.7780 | 8.2630 | 35.9670 | h1 | 1 | LIG | 0.033867 |
| 79 | H35 | 40.5540 | 7.3050 | 37.4100 | h1 | 1 | LIG | 0.033867 |
| 80 | H36 | 44.3860 | 8.5820 | 32.5770 | ha | 1 | LIG | 0.135000 |
| 81 | H37 | 44.6340 | 10.7960 | 34.5240 | ha | 1 | LIG | 0.130000 |

```
    82 H38          43.4650    10.6540    33.1790 ha          1 LIG       0.130000
@<TRIPOS>BOND
     1     1     2 1
     2     1     3 1
     3     1    25 1
     4     3     4 ar
     5     3     8 ar
     6     4     5 ar
     7     5     6 ar
     8     5    46 1
     9     6     7 ar
    10     6     9 1
    11     7     8 ar
    12     7    15 1
    13     9    10 2
    14     9    11 1
    15    11    12 1
    16    12    13 1
    17    12    14 1
    18    12    47 1
    19    13    48 1
    20    13    49 1
    21    13    50 1
    22    14    51 1
    23    14    52 1
    24    14    53 1
    25    15    16 2
    26    15    21 1
    27    16    17 1
    28    16    54 1
```

| | | | |
|---|---|---|---|
| 29 | 17 | 18 | 1 |
| 30 | 17 | 19 | 1 |
| 31 | 18 | 55 | 1 |
| 32 | 18 | 56 | 1 |
| 33 | 18 | 57 | 1 |
| 34 | 19 | 20 | ar |
| 35 | 19 | 21 | ar |
| 36 | 20 | 22 | ar |
| 37 | 20 | 58 | 1 |
| 38 | 21 | 24 | ar |
| 39 | 22 | 23 | ar |
| 40 | 22 | 59 | 1 |
| 41 | 23 | 24 | ar |
| 42 | 23 | 60 | 1 |
| 43 | 24 | 61 | 1 |
| 44 | 25 | 26 | ar |
| 45 | 25 | 30 | ar |
| 46 | 26 | 27 | ar |
| 47 | 26 | 31 | 1 |
| 48 | 27 | 28 | ar |
| 49 | 27 | 62 | 1 |
| 50 | 28 | 29 | ar |
| 51 | 28 | 33 | 1 |
| 52 | 29 | 30 | ar |
| 53 | 29 | 40 | 1 |
| 54 | 30 | 63 | 1 |
| 55 | 31 | 32 | 1 |
| 56 | 32 | 64 | 1 |
| 57 | 32 | 65 | 1 |
| 58 | 32 | 66 | 1 |

```
59 33 34 1
60 33 35 1
61 34 67 1
62 34 68 1
63 34 69 1
64 35 36 1
65 35 70 1
66 35 71 1
67 36 37 1
68 36 72 1
69 36 73 1
70 37 38 1
71 37 39 1
72 38 74 1
73 38 75 1
74 38 76 1
75 39 77 1
76 39 78 1
77 39 79 1
78 40 41 1
79 40 43 1
80 41 42 2
81 41 44 1
82 44 45 2
83 44 80 1
84 45 81 1
85 45 82 1
@<TRIPOS>SUBSTRUCTURE
 1 LIG 1 TEMP 0 **** **** 0 ROOT
```

**Table S7.** Definition of the ligand Entrectinib (in Mol2 format) including all of the charges and atom types to load into Leap.

```
@<TRIPOS>MOLECULE
UNN
  75   80    1    0    0
SMALL
bcc


@<TRIPOS>ATOM
      1 F          43.9290     1.1570    18.6650 f          0 UNN      -0.134900
      2 F1         47.1240     0.1840    21.8630 f          0 UNN      -0.134900
      3 O          46.4780     3.6460    20.4800 os         0 UNN      -0.415600
      4 O1         44.6930     9.9880    22.0740 o          0 UNN      -0.544100
      5 N          50.7480     9.2920    22.7290 nh         0 UNN      -0.638000
      6 N1         53.4470     9.1670    23.6410 n3         0 UNN      -0.728600
      7 N2         47.1330     7.1580    20.8170 nh         0 UNN      -0.747600
      8 N3         44.7880     7.7640    21.9940 n          0 UNN      -0.472400
      9 N4         41.4950     7.5350    21.4780 na         0 UNN      -0.003100
     10 N5         42.5290     8.2100    21.4900 nc         0 UNN      -0.525100
     11 C          47.9090     6.1250    20.0750 c3         0 UNN       0.205500
     12 C1         48.4290     4.9850    20.9600 c3         0 UNN      -0.122900
     13 C2         46.9880     5.4950    19.0140 c3         0 UNN      -0.122900
     14 C3         51.6530     8.2350    22.1990 c3         0 UNN       0.193800
     15 C4         51.2820    10.3800    23.5890 c3         0 UNN       0.193800
     16 C5         53.1550     8.5060    22.3800 c3         0 UNN       0.160800
     17 C6         52.8160    10.4760    23.6490 c3         0 UNN       0.160800
     18 C7         49.4810     9.2130    22.5230 ca         0 UNN       0.215600
     19 C8         47.2670     4.1710    21.5170 c3         0 UNN       0.126900
     20 C9         45.9080     4.6420    19.6720 c3         0 UNN       0.126900
     21 C10        47.5750     8.0950    21.5640 ca         0 UNN       0.225600
```

| | | | | | | | | |
|---|---|---|---|---|---|---|---|---|
| 22 | C11 | 48.8970 | 8.2300 | 21.8000 | ca | 0 | UNN | -0.263000 |
| 23 | C12 | 46.7370 | 9.0100 | 22.1000 | ca | 0 | UNN | -0.263600 |
| 24 | C13 | 48.6020 | 10.1070 | 23.0180 | ca | 0 | UNN | -0.236000 |
| 25 | C14 | 54.8840 | 9.2820 | 23.8470 | c3 | 0 | UNN | 0.156100 |
| 26 | C15 | 47.2820 | 10.0150 | 22.8080 | ca | 0 | UNN | -0.019000 |
| 27 | C16 | 45.3770 | 8.9950 | 21.9790 | c | 0 | UNN | 0.684700 |
| 28 | C17 | 43.4860 | 7.4580 | 21.8550 | cd | 0 | UNN | 0.421500 |
| 29 | C18 | 42.9770 | 6.2380 | 22.0890 | ca | 0 | UNN | -0.227900 |
| 30 | C19 | 41.6710 | 6.3330 | 21.8300 | ca | 0 | UNN | -0.046200 |
| 31 | C20 | 43.5020 | 5.0830 | 22.5110 | ca | 0 | UNN | -0.053000 |
| 32 | C21 | 42.6900 | 4.0170 | 22.6620 | ca | 0 | UNN | -0.114300 |
| 33 | C22 | 43.2520 | 2.7010 | 23.1590 | c3 | 0 | UNN | 0.008200 |
| 34 | C23 | 40.8410 | 5.2960 | 21.9570 | ca | 0 | UNN | -0.138000 |
| 35 | C24 | 41.3750 | 4.1360 | 22.3770 | ca | 0 | UNN | -0.094000 |
| 36 | C25 | 44.0730 | 1.9780 | 22.1130 | ca | 0 | UNN | -0.025300 |
| 37 | C26 | 45.2420 | 1.3960 | 22.4370 | ca | 0 | UNN | -0.180000 |
| 38 | C27 | 43.6450 | 1.8800 | 20.8410 | ca | 0 | UNN | -0.180000 |
| 39 | C28 | 44.3660 | 1.2340 | 19.9110 | ca | 0 | UNN | 0.155400 |
| 40 | C29 | 45.9740 | 0.7430 | 21.5210 | ca | 0 | UNN | 0.155400 |
| 41 | C30 | 45.5320 | 0.6650 | 20.2570 | ca | 0 | UNN | -0.209000 |
| 42 | H | 46.1540 | 7.1130 | 20.7190 | hn | 0 | UNN | 0.415800 |
| 43 | H1 | 45.3960 | 7.0000 | 22.1230 | hn | 0 | UNN | 0.334500 |
| 44 | H2 | 40.6170 | 7.8980 | 21.2190 | hn | 0 | UNN | 0.307700 |
| 45 | H3 | 48.7570 | 6.6240 | 19.6550 | h1 | 0 | UNN | 0.051700 |
| 46 | H4 | 48.9910 | 5.3970 | 21.7720 | hc | 0 | UNN | 0.058950 |
| 47 | H5 | 49.0500 | 4.3440 | 20.3700 | hc | 0 | UNN | 0.058950 |
| 48 | H6 | 46.5230 | 6.2740 | 18.4460 | hc | 0 | UNN | 0.058950 |
| 49 | H7 | 47.5770 | 4.8700 | 18.3760 | hc | 0 | UNN | 0.058950 |
| 50 | H8 | 51.4200 | 7.3180 | 22.6990 | h1 | 0 | UNN | 0.046450 |
| 51 | H9 | 51.4830 | 8.2020 | 21.1430 | h1 | 0 | UNN | 0.046450 |

```
  52 H10        50.9210   10.2220   24.5840 h1       0 UNN      0.046450
  53 H11        50.9450   11.2960   23.1510 h1       0 UNN      0.046450
  54 H12        53.4910    9.1300   21.5780 h1       0 UNN      0.040950
  55 H13        53.6610    7.5630   22.3790 h1       0 UNN      0.040950
  56 H14        53.0950   10.9880   24.5460 h1       0 UNN      0.040950
  57 H15        53.1470   11.0060   22.7810 h1       0 UNN      0.040950
  58 H16        47.6540    3.3650   22.1050 h1       0 UNN      0.052450
  59 H17        46.6570    4.8130   22.1180 h1       0 UNN      0.052450
  60 H18        45.3160    4.1790   18.9110 h1       0 UNN      0.052450
  61 H19        45.3010    5.2740   20.2860 h1       0 UNN      0.052450
  62 H20        49.5210    7.5170   21.3910 ha       0 UNN      0.140000
  63 H21        48.9600   10.8940   23.5810 ha       0 UNN      0.139000
  64 H22        55.3250    8.3070   23.8370 h1       0 UNN      0.035033
  65 H23        55.0730    9.7490   24.7910 h1       0 UNN      0.035033
  66 H24        55.3100    9.8740   23.0640 h1       0 UNN      0.035033
  67 H25        46.6630   10.7400   23.2030 ha       0 UNN      0.144000
  68 H26        44.5100    5.0090   22.7170 ha       0 UNN      0.137000
  69 H27        43.8740    2.8940   24.0080 hc       0 UNN      0.062200
  70 H28        42.4230    2.0720   23.4080 hc       0 UNN      0.062200
  71 H29        39.8350    5.3810   21.7430 ha       0 UNN      0.144000
  72 H30        40.7620    3.3120   22.4850 ha       0 UNN      0.135000
  73 H31        45.5830    1.4530   23.4090 ha       0 UNN      0.154500
  74 H32        42.7450    2.3100   20.5750 ha       0 UNN      0.154500
  75 H33        46.0900    0.1660   19.5470 ha       0 UNN      0.166000
@<TRIPOS>BOND
     1     1    39 1
     2     2    40 1
     3     3    19 1
     4     3    20 1
     5     4    27 2
```

| | | | |
|---|---|---|---|
| 6 | 5 | 14 | 1 |
| 7 | 5 | 15 | 1 |
| 8 | 5 | 18 | 1 |
| 9 | 6 | 16 | 1 |
| 10 | 6 | 17 | 1 |
| 11 | 6 | 25 | 1 |
| 12 | 7 | 11 | 1 |
| 13 | 7 | 21 | 1 |
| 14 | 7 | 42 | 1 |
| 15 | 8 | 27 | 1 |
| 16 | 8 | 28 | 1 |
| 17 | 8 | 43 | 1 |
| 18 | 9 | 10 | 1 |
| 19 | 9 | 30 | 1 |
| 20 | 9 | 44 | 1 |
| 21 | 10 | 28 | 2 |
| 22 | 11 | 12 | 1 |
| 23 | 11 | 13 | 1 |
| 24 | 11 | 45 | 1 |
| 25 | 12 | 19 | 1 |
| 26 | 12 | 46 | 1 |
| 27 | 12 | 47 | 1 |
| 28 | 13 | 20 | 1 |
| 29 | 13 | 48 | 1 |
| 30 | 13 | 49 | 1 |
| 31 | 14 | 16 | 1 |
| 32 | 14 | 50 | 1 |
| 33 | 14 | 51 | 1 |
| 34 | 15 | 17 | 1 |
| 35 | 15 | 52 | 1 |

| | | |
|---|---|---|
| 36 | 15 | 53 1 |
| 37 | 16 | 54 1 |
| 38 | 16 | 55 1 |
| 39 | 17 | 56 1 |
| 40 | 17 | 57 1 |
| 41 | 18 | 22 ar |
| 42 | 18 | 24 ar |
| 43 | 19 | 58 1 |
| 44 | 19 | 59 1 |
| 45 | 20 | 60 1 |
| 46 | 20 | 61 1 |
| 47 | 21 | 22 ar |
| 48 | 21 | 23 ar |
| 49 | 22 | 62 1 |
| 50 | 23 | 26 ar |
| 51 | 23 | 27 1 |
| 52 | 24 | 26 ar |
| 53 | 24 | 63 1 |
| 54 | 25 | 64 1 |
| 55 | 25 | 65 1 |
| 56 | 25 | 66 1 |
| 57 | 26 | 67 1 |
| 58 | 28 | 29 1 |
| 59 | 29 | 30 ar |
| 60 | 29 | 31 ar |
| 61 | 30 | 34 ar |
| 62 | 31 | 32 ar |
| 63 | 31 | 68 1 |
| 64 | 32 | 33 1 |
| 65 | 32 | 35 ar |

```
    66    33    36 1
    67    33    69 1
    68    33    70 1
    69    34    35 ar
    70    34    71 1
    71    35    72 1
    72    36    37 ar
    73    36    38 ar
    74    37    40 ar
    75    37    73 1
    76    38    39 ar
    77    38    74 1
    78    39    41 ar
    79    40    41 ar
    80    41    75 1
@<TRIPOS>SUBSTRUCTURE
     1 UNN         1 TEMP              0 ****  ****    0 ROOT
```

**References:**


1. Z. Fu, X. Li, K. M. Merz Jr, *J. Comput. Chem.* 2011, **32**, 2587−2597.
2. Y. Zhao, D. G. Truhlar, *Theor. Chem. Acc.,* 2008, **120**, 215−241.
3. J. W. Vant, S.-L. J. Lahey, K. Jana, M. Shekhar, D. Sarkar, B. H. Munk, U. Kleinekathöfer, S. Mittal, C. Rowley, A. Singharoy, *J. Chem. Inf. Model.,* 2020, **60**, 2591−2604.
